# Computational analysis of microarray data of *Arabidopsis thaliana* challenged with *Alternaria brassicicola* for identification of key genes in *Brassica*

**DOI:** 10.1186/s43141-020-00032-y

**Published:** 2020-07-01

**Authors:** Rajesh Kumar Pathak, Mamta Baunthiyal, Dinesh Pandey, Anil Kumar

**Affiliations:** 1 Department of Biotechnology, Govind Ballabh Pant Institute of Engineering & Technology, Pauri Garhwal, Uttarakhand 246194 India; 2 Department of Molecular Biology & Genetic Engineering, College of Basic Sciences & Humanities, G. B. Pant University of Agriculture & Technology, Pantnagar, Uttarakhand 263145 India; 3 Rani Lakshmi Bai Central Agricultural University, Jhansi, Uttar Pradesh 284003 India

**Keywords:** Microarray data analysis, GO analysis, Network analysis, Hubs, Oilseeds, *Brassica*

## Abstract

**Background:**

*Alternaria* blight, a recalcitrant disease caused by *Alternaria brassicae* and *Alternaria brassicicola*, has been recognized for significant losses of oilseed crops especially rapeseed-mustard throughout the world. Till date, no resistance source is available against the disease; hence, plant breeding methods cannot be used to develop disease-resistant varieties. Therefore, in the present study, efforts have been made to identify resistance and defense-related genes as well as key components of *JA-SA-ET*-mediated pathway involved in resistance against *Alternaria brasscicola* through computational analysis of microarray data and network biology approach. Microarray profiling data from wild type and mutant *Arabidopsis* plants challenged with *Alternaria brassicicola* along with control plant were obtained from the Gene Expression Omnibus (GEO) database. The data analysis, including DEGs extraction, functional enrichment, annotation, and network analysis, was used to identify genes associated with disease resistance and defense response.

**Results:**

A total of 2854 genes were differentially expressed in WT9C9; among them, 1327 genes were upregulated and 1527 genes were downregulated. A total of 1159 genes were differentially expressed in JAM9C9; among them, 809 were upregulated and 350 were downregulated. A total of 2516 genes were differentially expressed in SAM9C9; among them, 1355 were upregulated and 1161 were downregulated. A total of 1567 genes were differentially expressed in ETM9C9; among them, 917 were upregulated and 650 were downregulated. Besides, a total of 2965 genes were differentially expressed in contrast WT24C24; among them, 1510 genes were upregulated and 1455 genes were downregulated. A total of 4598 genes were differentially expressed in JAM24C24; among them, 2201 were upregulated and 2397 were downregulated. A total of 3803 genes were differentially expressed in SAM24C24; among them, 1819 were upregulated and 1984 were downregulated. A total of 4164 genes were differentially expressed in ETM24C24; among them, 1895 were upregulated and 2269 were downregulated. The upregulated genes of *Arabidopsis thaliana* were mapped and annotated with CDS sequences of *Brassica rapa* obtained from PlantGDB database. Additionally, PPI network of these genes were constructed to investigate the key components of hormone-mediated pathway involved in resistance during pathogenesis.

**Conclusion:**

The obtained information from present study can be used to engineer resistance to *Alternaria* blight caused by *Alternaria brasscicola* through molecular breeding or genetic manipulation-based approaches for improving *Brassica* oilseed productivity.

## Background

*Alternaria* blight, a recalcitrant disease caused by *Alternaria brassicae* and *Alternaria brassicicola*, has been recognized for significant losses of oilseed crops especially rapeseed-mustard throughout the world. Till date, no resistance source is available against the disease; hence, plant breeding methods cannot be used to develop disease-resistant varieties. Oilseed crops especially *Brassica* (rapeseed-mustard) play a critical role in the Indian agricultural economy, next to food grains, in terms of area, production, and value. It is grown in 53 countries across the six continents, with India being the world’s second largest grower after China [1, 2]. Despite that, India has to import large amount of edible oils from other countries to meet its domestic demands [3]. In future, the demand for oilseed production is expected to extensively increase due to increase in population and income. The only way to increase oilseed productivity is to protect mustard crops from the attack of various biotic and abiotic stresses [4].

Fungi and oomycete are the main threats causing major losses in oilseed crops; more than thirty diseases are incurred in mustard crops in India [5, 6]. *Alternaria* blight, caused by *Alternaria brassicae* and *Alternaria brassicicola*, holds major importance based on the economic yield losses in *Brassica* crops [6, 7]. The yield losses due to *Alternaria* blight disease have been estimated to range from 35 to 46% in India and up to 70% in the world with no demonstrated source of transferable resistance in any of the hosts [8, 9]. Disease management strategies employing fungicidal chemicals are not only environmentally hazardous but also inadequate to control the disease caused by *Alternaria brasscicola*. Quick evolution through genetic variations of new pathogenic strains has further been problematical for breeders to develop resistance in crop plants. *Alternaria* is a necrotrophic fungal pathogen which produces lesions on leaves, siliquae, and stems influencing quantity as well as quality of seed by diminishing oil content, size, and color [10, 11].

Phytohormones affect several aspects of growth and differentiation in crop plants and are involved in both abiotic and biotic stress responses in plants. Among the plant hormones, jasmonic acid (JA), salicylic acid (SA), and ethylene (ET), which are known for differentially controlling defense responses against biotrophic and necrotrophic pathogens, are recognized as the immunity hormones [12, 13]. The accumulation of these hormones triggers the activation of a cascade of defense-signaling pathways. However, the final outcome of the defense response is greatly influenced by the production, timing, and composition of the hormonal blend produced [14–18]. Although there are exceptions, in general, it can be stated that SA-dependent defenses and JA/ET-dependent defenses participate in defense against biotrophic and necrotrophic pathogens and against insect herbivores respectively [7, 19–21]. Jasmonic acid (JA)-dependent defense signaling pathway has been reported to restrict the growth of necrotrophic fungal pathogens [2, 22, 23]. The expression of some MAP kinases has been associated with increase in JA level in plants and JA-dependent genes. For example, expression of MAPK4 is linked with induction of JA-dependent genes/proteins, and MAPK6 which triggers the basal defense is also activated by JA [7, 24]. The downregulation of MAPK4 as observed during pathogenesis of *Alternaria* blight is an indication of decrease in JA-dependent defense against the pathogen. Since the pathogen is a hemibiotrophic which uses both biotrophic and necrotrophic mode of infection, hence, it was thought that downregulation of JA-mediated defense could facilitate necrotrophic colonization of pathogen on host. However, no information is available about intricacy of such signaling cascades involved in the pathogenesis, though some evidences of antagonism of *Alternaria* toxin and zeatin are reported in this system [25]. Plant breeders are unable to develop resistance against *Alternaria* blight due to lack of knowledge of resistant genes linked with defense responses. Although some progress has been made in recent years to understand the molecular basis of pathogenesis of *Alternaria* blight, the target molecules affected by disease is not identified [7].

In the view of the above facts, there is a need of genomics- and bioinformatics-based approaches to decipher the complexity of signaling cascades through analysis of available microarray data of host-pathogen interaction for identification of defense-related gene(s) involved in hormone-mediated resistance which can be utilized for the development of disease-resistant *Brassica* crops through genetic manipulation of key candidate gene(s) or by utilizing molecular breeding approaches for sustainable agriculture.

## Methods

### Source of DNA microarray data

The microarray datasets, GSE50526 with GPL198 [ATH1-121501] Affymetrix *Arabidopsis* ATH1 Genome array platform, were obtained from the Gene Expression Omnibus (GEO) database of the National Center for Biotechnology Information [26]. The data samples were obtained from the *Arabidopsis* leaves which were challenged with the *Alternaria brassicicola* infection at 9 and 24 h. The GSE50526 dataset contains 29 leaf samples of wild type and JA-SA-ET mutant plant that is also challenged with the infection of *Alternaria brassicicola* along with the control.

### Pre-processing of raw data

All 29 sample files (.CEL files) were subjected to the R software library (version 3.4.0) (https://www.r-project.org/). The Affy library of Bioconductor was used to read CEL files. Subsequently, simpleaffy library was used to check the quality of raw data (https://www.bioconductor.org/). GCRMA algorithm was applied for normalization and summarization of the probes [27]. The obtained normalized expression values were utilized for further analysis.

### Screening and annotation of differentially expressed genes

The linear modeling approach was employed for screening of differentially expressed genes (DEGs). The limma library in R/Bioconductor was used to build the linear models and contrasts of interest [28]. To obtain DEGs, moderated *t* statistic has been applied. The multiplicity of testing was done using the Benjamini and Hochberg (BH) correction adjusted for false discovery rate (FDR). The threshold adjusted *p* value was set as < 0.05, and fold-change threshold was set to > 1.5. The decideTests function was implemented to fetch out up- and downregulated probes present in each contrasts. The library org.At.tair.db, ath1121501.db, and annotate was used to get Gene Symbol, EntrezID, and TAIR accession number of up- and downregulated probes [29, 30].

### Enrichment analysis of the DEGs

The gene ontology (GO) enrichment analysis, i.e., biological process, molecular function, and cellular component of up- and downregulated genes were performed by GeneCodis (http://genecodis.cnb.csic.es/). Besides, pathway analysis was also done using Kyoto Encyclopedia of Genes and Genome (KEGG) by the same tool [31–33]. The threshold value was set at *p* < 0.05.

### Mapping of identified upregulated gene(s) sequences in *Arabidopsis thaliana* on *Brassica rapa*

All annotated upregulated gene sequences involved in defense response to fungi of each contrasts at 9 and 24 h have been taken, merged to prepare a single text file for every contrasts, i.e., WTC, JAMC, SAMC, and ETMC. Many genes were found to be upregulated in both conditions, i.e., 9 and 24 h in each contrast during analysis; therefore, duplicate sequences were removed, and the rest are considered for analysis in such condition. The complete CDS protein and nucleotide sequences of *Arabidopsis thaliana* each gene were downloaded from TAIR (https://www.arabidopsis.org/) database through batch download using accession numbers, whereas available CDS sequences of *Brassica* from *Brassica rapa* genome (*n* = 41019; 14.52 MB) were downloaded from BrGDB, part of the PlantGDB database (www.plantgdb.org) (accessed on 21 July, 2017). These sequences were used to construct a local database of *B. rapa* CDS sequence. Further, the retrieved sequences of upregulated genes of *Arabidopsis thaliana* form TAIR were taken as a query to perform local BLAST search against constructed local database of the *B. rapa* sequences to determine the closeness among them [34]. The top BLAST hits of *B. rapa* sequences that pose higher identity and lower e-value with *A. thaliana* sequences were taken for further investigations.

### Characterization and comparative analysis of identified up-regulated gene(s) through molecular phylogeny and domain prediction

A single text file holding *A. thaliana* and its corresponding *B. rapa* sequences was created for each contrast taken in the study. Multiple sequence alignment was performed using CLUSTALX [35]. The molecular phylogeny was done by using aligned files to build a phylogenetic tree using NJ methods to visualize the relatedness between sequences using TreeView and iTOL [36, 37]. The presence of conserved domains in each sequence of every contrast was also determined via Conserved Domain Database (CDD) at the National Center for Biotechnology Information for characterization of gene(s) involved in disease resistance and defense responses against *Alternaria* blight in *Brassica spp* [38].

### Protein-protein interactions (PPIs) network construction and analysis of upregulated DEGs

**Fig. 1:**
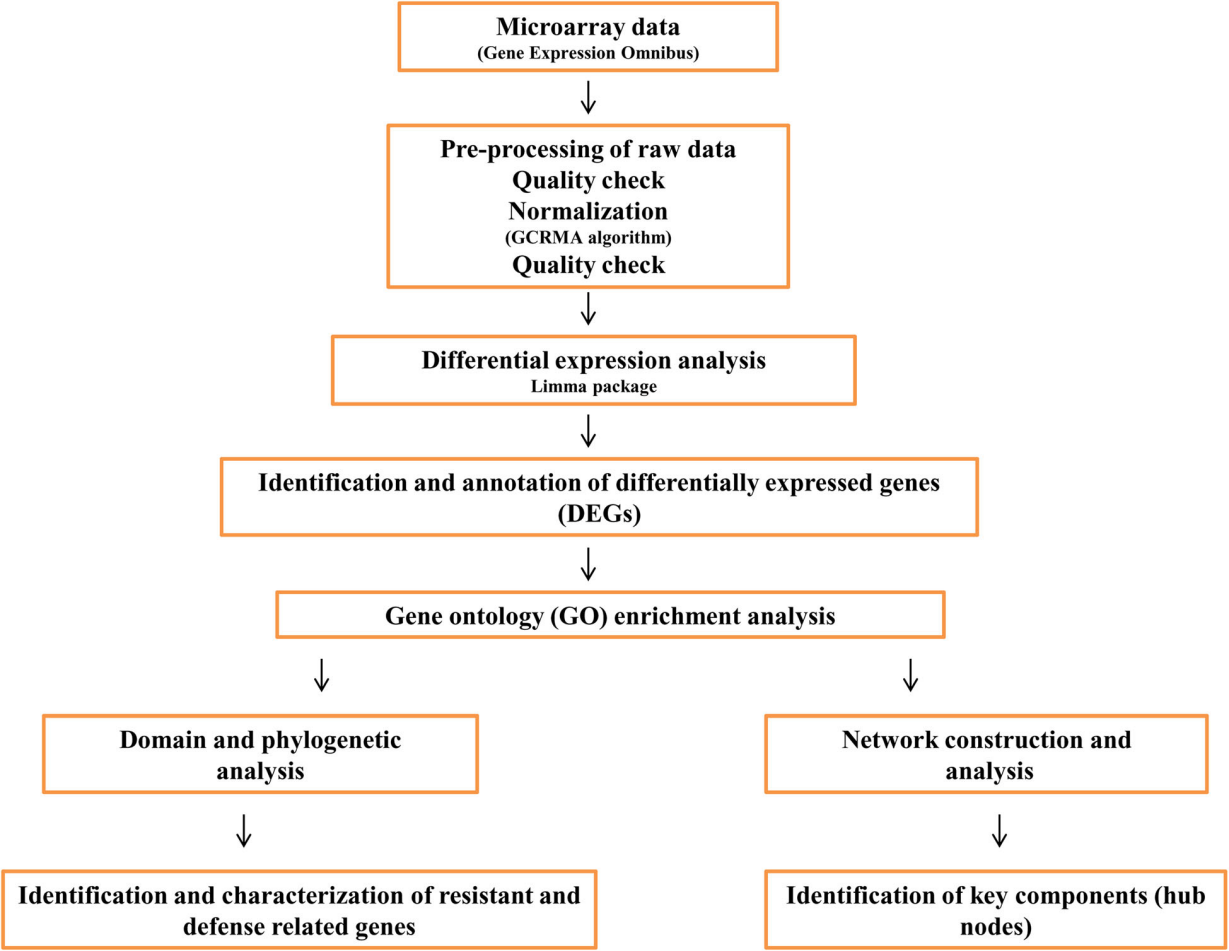
Overview of the data analysis workflow conducted in this study

The protein sequences of upregulated genes retrieved from TAIR were used to obtain PPIs network for JAMC, SAMC, and ETMC contrast from STRING (Search Tool for Retrieval of Interacting Genes/Protein) database [39]. STRING holds information about the experimental and predicted PPI obtained from scientific literature, which are based on their co-expression, neighborhood, co-occurrence, and gene fusion experimentation. The extended network for selected contrast was constructed based on high confidence score, which is considered as valid link. The obtained networks were visualized and analyzed topologically by Cytoscape 3.4.0 (http://www.cytoscape.org/) using Network Analyzer 3.3.1 to identify key components involved in resistance during pathogenesis of *Alternaria* blight with respect to JA-, SA-, and ET-mediated signaling pathway [40, 41]. A brief workflow is provided in Fig. 1 on the data and methods used in this analysis.

## Results

### Identification of upregulated and downregulated DEGs in wild and mutant plants of *A. thaliana* challenged with *Alternaria brassicicola*

In the biological systems, downregulation is the mechanism by which a cell, in response to an external stimulus, decreases the amount of a cellular component, such as RNA or protein. Besides, the complementary mechanism involving increase in these components is called upregulation which plays tremendous role during plant-pathogen interactions. After pre-processing of data, 22,810 probes were obtained on the basis of the cutoff criteria. A total of 1327 upregulated and 1527 downregulated probes were identified in wild-type pathogen-treated plant compared with the control at 9 h (WT9C9) whereas 1510 upregulated and 1455 downregulated probes were identified at 24 h (WT24C24); 809 upregulated and 350 downregulated at 9 h (JAM9C9) whereas 2201 upregulated and 2397 downregulated probes at 24 h (JAM24C24) were identified in jasmonic acid mutant plant challenged with pathogen compared with control plant; 1355 upregulated and 1161 downregulated at 9 h (SAM9C9) whereas 1819 upregulated and 1984 downregulated probes at 24 h (SAM24C24) were identified in salicylic acid mutant plant challenged with pathogen compared with control. Besides, 917 upregulated and 650 downregulated probes at 9 h (ETM9C9) as well as 1895 upregulated and 2269 downregulated probes at 24 h (ETM24C24) were also identified in ethylene mutant plant challenged with pathogen compared with the control plant (Table 1). The list of top-ten up- and downregulated genes in each contrasts are listed in Supplementary Table 1-16.

**Table 1:** Analysis of DEGs triggered during pathogenesis of *Alternaria* blight disease in comparison of wild-type pathogen-treated plant with control plant (WTC), jasmonic acid mutant pathogen-treated plant with control (JAMC), salicylic acid mutant pathogen-treated plant with control (SAMC), ethylene mutant-treated plant with control (ETMC) at 9 h and 24 h after *Alternaria brassicicola* infection on *Arabidopsis thaliana*

Contrasts	Upregulation	Downregulation	Total DEGs
WT9C9	1327	1527	2854
JAM9C9	809	350	1159
SAM9C9	1355	1161	2516
ETM9C9	917	650	1567
WT24C24	1510	1455	2965
JAM24C24	2201	2397	4598
SAM24C24	1819	1984	3803
ETM24C24	1895	2269	4164

Genes that respond to the conditions have been identified by comparing their expression levels in treatment and control samples. Out of total DEGs, only annotated probes having unique accession number were used for construction of Venn diagram because many probes code the same genes. In the present study, 1312 up and 1506 downregulated probes were annotated in WT9C9 whereas 1497 up and 1436 downregulated probes were annotated in WT24C24; 801 up and 342 downregulated probes were annotated in JAM9C9 whereas 2179 up and 2367 downregulated probes were annotated in JAM24C24; 1336 up and 1143 downregulated probes were annotated in SAM9C9 whereas 1805 up and 1954 downregulated probes were annotated in SAM24C24; 905 up and 642 downregulated probes were annotated in ETM9C9 whereas 1875 up and 2235 downregulated probes were annotated in ETM24C24. During analysis, NHL10 and HCHIB were identified as important genes which are involved in defense responses during pathogenesis of *Alternaria* blight in *Arabidopsis thaliana*.

Venn diagrams can be used for several purposes, such as comparing different lists of genes or proteins to define and represent similarity and differences in two dimensions. During Venn diagram construction and analysis, it was found that 152, 42, 220, and 40 genes are unique in WT9C9, JAM9C9, SAM9C9, ETM9C9, respectively. Besides, 602 are found common in WT9C9, JAM9C9, SAM9C9, and ETM9C9; 58 genes are found common in WT9C9, JAM9C9 and SAM9C9; 40 are common in WT9C9, JAM9C9, and ETM9C9; 151 are common in WT9C9, SAM9C9, and ETM9C9; 7 are common in JAM9C9, SAM9C9, and ETM9C9; 15 are common inWT9C9 and JAM9C9; 256 are common between WT9C9 and SAM9C9; 29 are common between WT9C9 and ETM9C9; 17 are common between JAM9C9 and SAM9C9; 13 are common in JAM9C9 and ETM9C9; and 15 genes are common between SAM9C9 and ETM9C9 at 9 h during upregulation. Upon analysis of downregulation of genes at 9 h, it was found that 624, 53, 425, and 67 genes are unique in WT9C9, JAM9C9, SAM9C9, and ETM9C9, respectively. Besides, 186 genes are common among WT9C9, JAM9C9, SAM9C9, and ETM9C9; 37 are common among WT9C9, JAM9C9, and SAM9C9; 24 are common among WT9C9, JAM9C9, and ETM9C9; 185 are common among WT9C9, SAM9C9, and ETM9C9; 6 are common in JAM9C9, SAM9C9, and ETM9C9; 21 are common among WT9C9 and JAM9C9; 273 are common among WT9C9 and SAM9C9; 152 are common among WT9C9 and ETM9C9; 12 are common among JAM9C9 and SAM9C9; 3 are common among JAM9C9 and ETM9C9; and 18 genes are common among SAM9C9 and ETM9C9.

**Fig. 2:**
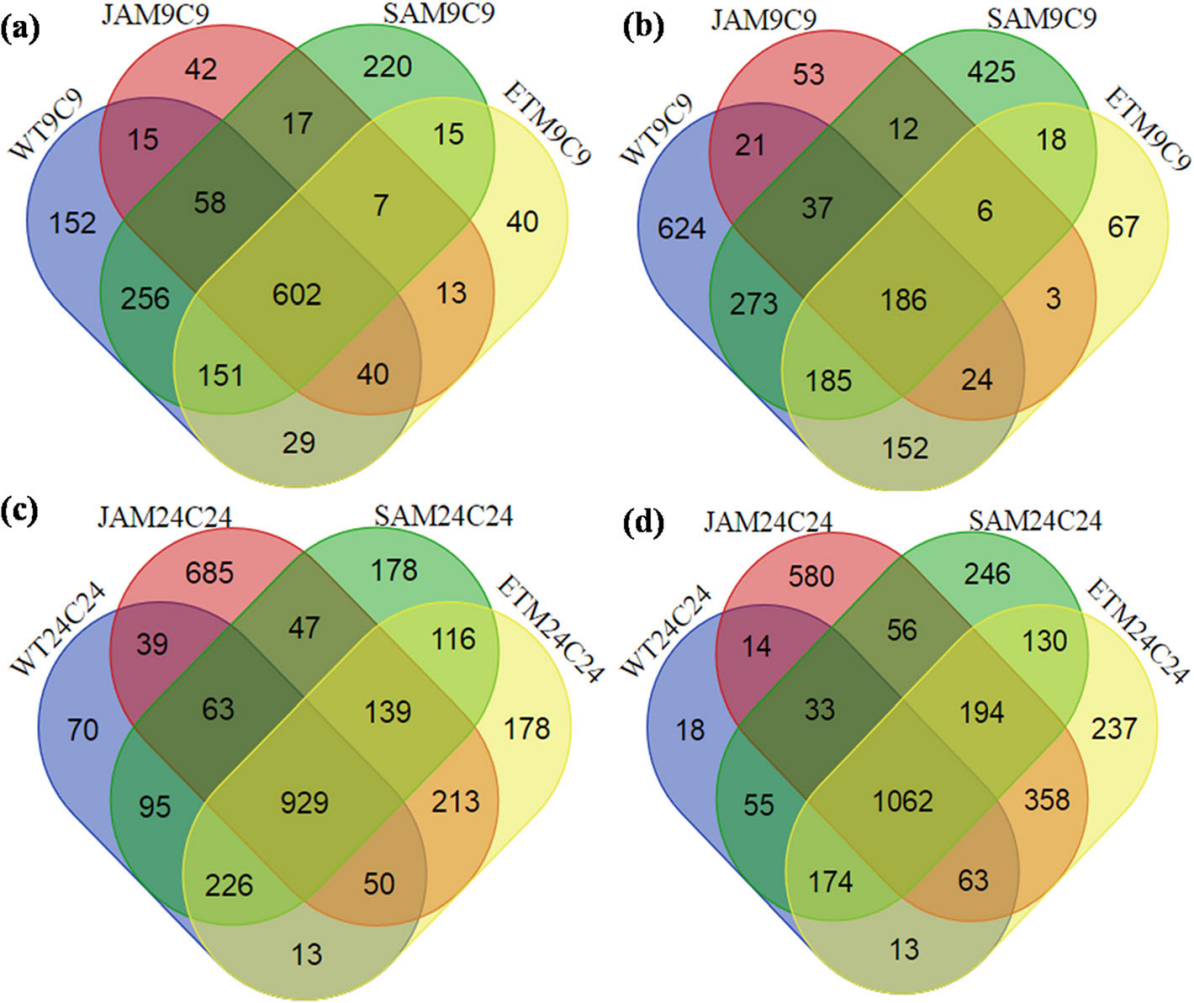
Venn diagram of unique and commonly expressed genes at **a** 9 h upregulation. **b** 9 h downregulation. **c** 24 h upregulation. **d** 24 h downregulation

Among the upregulated genes at 24 h of treatment, it was found that 70, 685, and 178 genes are unique in WT24C24, JAM24C24, SAM24C24, and ETM24C24 respectively. Besides, 929 genes are common among WT24C24, JAM24C24, SAM24C24, and ETM24C24; 63 are common among WT24C24, JAM24C24, and SAM24C24; 50 are common among WT24C24, JAM24C24, and ETM24C24; 226 are common among WT24C24, SAM24C24, and ETM24C24; 139 are common among JAM24C24, SAM24C24, and ETM24C24; 39 are common among WT24C24 and JAM24C24; 95 are common among WT24C24 and SAM24C24; 13 are common among WT24C24 and ETM24C24; 47 are common among JAM24C24 and SAM24C24; 213 are common among JAM24C24 and ETM24C24; and 116 genes are found common among SAM24C24 and ETM24C24. In case of downregulation, 18 genes are found unique in WT24C24, 580 are unique in JAM24C24, 246 are unique in SAM24C24, and 237 are unique in ETM24C24. Besides, 1062 genes are common among WT24C24, JAM24C24, SAM24C24, and ETM24C24; 33 genes are common among WT24C24, JAM24C24, and SAM24C24; 63 are common among WT24C24, JAM24C24, and ETM24C24; 174 are common among WT24C24, SAM24C24, and ETM24C24; 194 are common among JAM24C24, SAM24C24, and ETM24C24; 14 are common among WT24C24 and JAM24C24; 55 are common among WT24C24 and SAM24C24; 13 genes are common among WT24C24 and ETM24C24; 56 are common among JAM24C24 and SAM24C24; 358 are common among JAM24C24 and ETM24C24; and 130 are found common among SAM24C24 and ETM24C24. All the unique and common DEGs of each set are shown in Fig. 2.

### Enrichment analysis of up- and downregulated DEGs

Further biological knowledge were fetched from the list of DEGs that are known to perform biological process or involved in different key regulatory networks with respect to plant-pathogen interactions. The enrichment analysis of each contrast were done in terms of gene ontology (GO) analysis, i.e., biological process, molecular function, and cellular components as well as KEGG analysis for decoding the role of DEGs in plant systems during pathogenesis of *Alternaria brassicicola*.

In contrast WT9C9 during upregulation, the significant GO term for biological function was protein phosphorylation (GO 0006468) whereas 38 genes were detected which are involved in the defense response to fungus (GO 0050832), for molecular function was protein binding (GO 0005515), and for cellular component was plasma membrane (GO 0005886). In downregulation condition of WT9C9, the significant GO term for biological process was metabolic process (GO 0008152), for molecular function was ATP binding (GO 0005524), and for cellular component was chloroplast (GO 0009507). In upregulation condition of JAM9C9, the significant GO term for biological process was protein phosphorylation (GO 0006468), whereas 35 genes were involved in defense response to fungus (GO 0050832), for molecular function was kinase activity (GO 0016301), for cellular component was plasma membrane (GO 0005886). In case of downregulation of JAM9C9, the significant GO term for biological process was metabolic process (GO 0008152), for molecular function was DNA binding (GO 0003677), for cellular component was chloroplast (GO 0009507). In upregulation condition of SAM9C9, the significant GO term for biological process was regulation of transcription, DNA dependent (GO:0006355), whereas 34 genes were involved in defense response to fungus, for molecular function was sequence-specific DNA binding transcription factor activity (GO 0003700), for cellular component was plasma membrane (GO 0005886). Besides, in downregulation condition of SAM9C9, the significant GO term for biological process was protein phosphorylation (GO 0006468), for molecular function was sequence-specific DNA binding transcription factor activity (GO 0003700), and for cellular component was chloroplast (GO 0009507). In upregulation condition of ETM9C9, the significant GO term for biological process was response to chitin (GO 0010200), whereas 37 genes were involved in defense response to fungus (GO 0050832), for molecular function was kinase activity (GO 0016301), for cellular component was plasma membrane (GO 0005886). Besides, in downregulation of ETM9C9, the significant GO term for biological process was proteolysis (GO 0006508), for molecular function was protein binding (GO 0005515), and cellular component was chloroplast (GO 0009507). In contrast WT24C24 during upregulation, the significant GO term for biological function was metabolic process (GO 0008152) whereas 34 genes were detected which are involved in the defense response to fungus (GO 0050832), for molecular function was protein binding (GO 0005515), for cellular component was cytosol (GO 0005829). In downregulation condition of WT24C24, the significant GO term for biological process was metabolic process (GO 0008152), for molecular function was catalytic activity (GO 0003824), and for cellular component was chloroplast (GO 0009507). In upregulation condition of JAM24C24, the significant GO term for biological process was metabolic process (GO 0008152), whereas 48 genes were involved in defense response to fungus (GO 0050832), for molecular function was protein binding (GO 0005515), for cellular component was cytosol (GO 0005829). In case of downregulation of JAM24C24, the significant GO terms for biological process, molecular function, and cellular components were translation (GO 0006412), structural constituent of ribosome (GO 0003735), and chloroplast (GO 0009507), respectively. In up-regulation condition of SAM24C24, the significant GO terms for biological process, molecular function, and cellular component were metabolic process (GO 0008152), whereas 33 genes were involved in defense response to fungus, protein binding (GO 0005515), and cytosol (GO 0005829), respectively. Besides, in downregulation condition of SAM24C24, the significant GO terms for biological process, molecular function, and cellular components were metabolic process (GO 0008152), catalytic activity (GO 0003824), and chloroplast (GO:0009507), respectively. In upregulation condition of ETM24C24, the significant GO terms for biological process, molecular function, and cellular component were metabolic process (GO 0008152), whereas 39 genes were involved in defense response to fungus (GO 0050832), protein binding (GO 0005515), and cytosol (GO 0005829), respectively. Besides, in downregulation of ETM24C24, the significant GO terms for biological process, molecular function, and cellular component were translation (GO 0006412), catalytic activity (GO 0003824), and chloroplast (GO 0009507), respectively.

Pathways analysis is a useful tool for understanding the interrelationship between different biological components to recognize key pathway. The KEGG pathway enrichment analysis was done to further evaluate up- and downregulated genes involved in different biological function. The significant pathway term was sorted based on *P* value. Our analysis revealed that amino sugar and nucleotide sugar metabolism (KEGG 00520) was the most significant pathway of upregulated condition in WT9C9. While, in downregulated condition of WT9C9, starch and sucrose metabolism (KEGG 00500) was the most significant pathway; biosynthesis of secondary metabolites (KEGG 01110) was the significant pathway in JAM9C9 upregulated, whereas plant hormone signal transduction (KEGG 04075) was in downregulated condition of JAM9C9. Amino sugar and nucleotide sugar metabolism (KEGG 00520) was the significant pathway in SAM9C9 upregulated, whereas glycosphingolipid biosynthesis—globo series (KEGG 00603) was in downregulated condition of SAM9C9; glutathione metabolism (KEGG 00480) was the significant pathway in ETM9C9 upregulated, whereas peroxisome (KEGG 04146) was in downregulated condition of ETM9C9. Oxidative phosphorylation (KEGG 00190) was the most significant pathway of upregulated condition in WT24C24. While in downregulated condition of WT24C24, starch and sucrose metabolism (KEGG 00500) was the most significant pathway; amino sugar and nucleotide sugar metabolism (KEGG 00520) was the significant pathway in JAM24C24 upregulated, whereas starch and sucrose metabolism (KEGG 00500) was in down-regulated condition of JAM24C24; starch and sucrose metabolism (KEGG 00500) was the significant pathway of upregulated and downregulated condition in SAM24C24 and ETM24C24. Furthermore, plant-hormone signal transduction (KEGG 04075) and plant-pathogen interaction (KEGG 04626) were revealed to be highly enriched in upregulated conditions. Therefore, the plant hormone-based signaling network plays significant role during pathogenesis and triggering defense to plant systems towards pest and pathogens.

### Identification and characterization of genes in *Brassica* based on upregulated DEGs triggered during resistance against *A. brassicicola* in *A. thaliana*

Based on the gene ontology analysis, the genes triggered in *Arabidopsis thaliana* during resistance to fungal pathogen (upregulated) have been taken for further analysis. A total of 47, 52, 45, and 49 unique genes were chosen from WTC, JAMC, SAMC, and ETMC respectively at 9 and 24 h. Out of these, 41, 42, 40, and 42 genes were annotated in WTC, JAMC, SAMC, and ETMC, respectively through BLAST analysis against constructed local database of *Brassica rapa* based on bit score, identity, and e-value (Supplementary table 17-20).

**Fig. 3:**
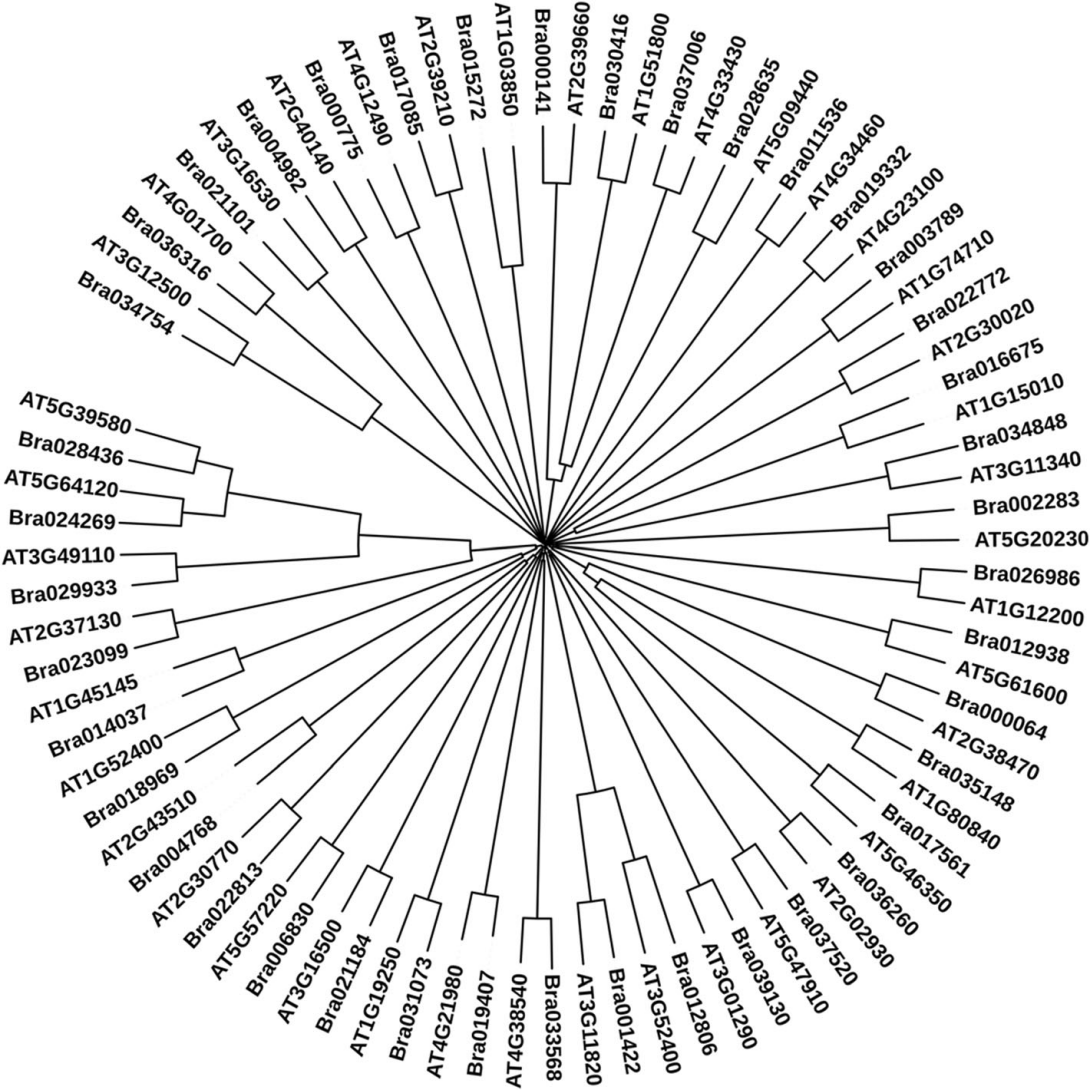
Neighbor-Joining tree was constructed to determine the relationship among *Arabidopsis* and *Brassica* sequences involved in defense response against fungi extracted from contrast WTC and BrGDB

**Fig. 4:**
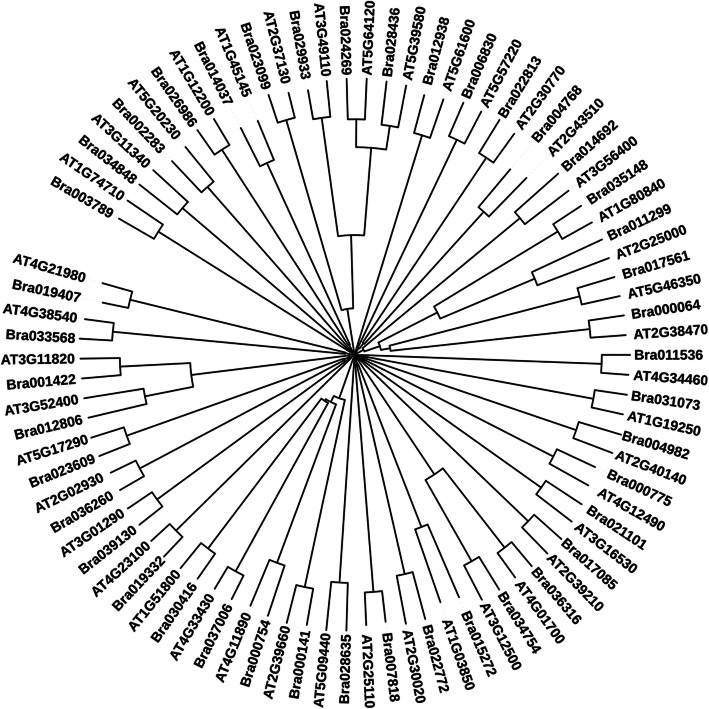
Neighbor-Joining tree was constructed to determine the relationship among *Arabidopsis* and *Brassica* sequences involved in defense response against fungi extracted from contrast JAMC and BrGDB

**Fig. 5:**
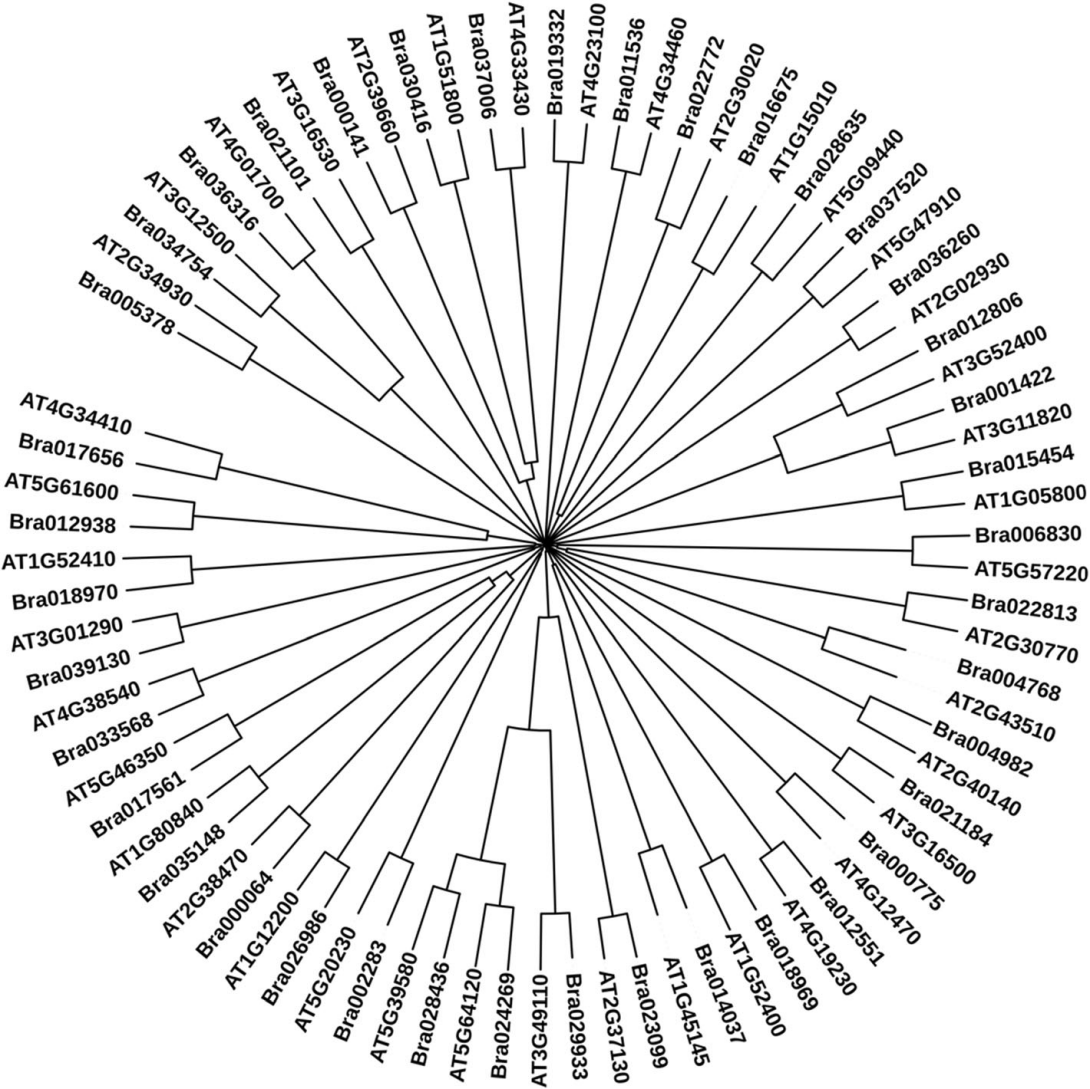
Neighbor-Joining tree was constructed to determine the relationship among *Arabidopsis* and *Brassica* sequences involved in defense response against fungi extracted from contrast SAMC and BrGDB

**Fig. 6:**
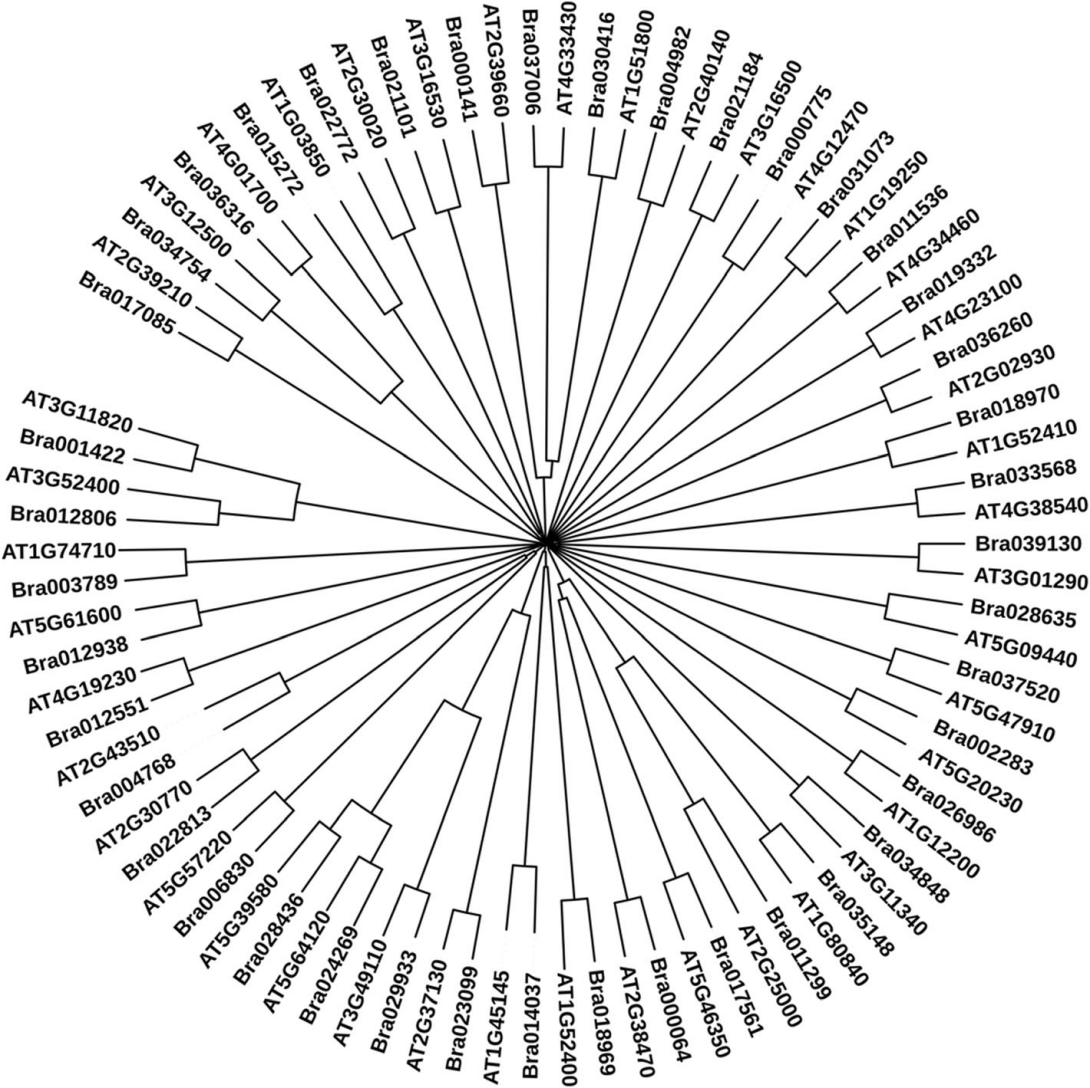
Neighbor-Joining tree was constructed to determine the relationship among *Arabidopsis* and *Brassica* sequences involved in defense response against fungi extracted from contrast ETMC and BrGDB

The identified sequences of *Brassica rapa* were further subjected to domain prediction for functional characterization and molecular phylogeny analysis with *Arabidopsis* for their relatedness prediction among them. The number of predicted domain and their positions along with short names for WTC, JAMC, SAMC, and ETMC are given in Tables 2, 3, 4, and 5 respectively. Based on obtained results, it can be interpreted that they might be involved in disease resistance and defense responses during pathogenesis. To examine the evolutionary relationship among identified *Arabidopsis* sequences with respect to similar *Brassica rapa* sequences obtained through BLAST analysis, phylogenetic tree for WTC, JAMC, SAMC, and ETMC were constructed to determine the relationship among them (Figs. 3, 4, 5, 6).

**Table 2:** Predicted conserved domains in identified upregulated *Brassica rapa* sequences with their positions under contrast WTC

S.N.	*B. rapa* accession	No. of conserved domains	From	To	Predicted domain (short name)
1.	Bra006830	1	26	485	p450 superfamily
2.	Bra035148	1	147	205	WRKY
3.	Bra017561	1	178	235	WRKY
4.	Bra036260	2	96 4	212 78	GST_C_Phi GST_N_Phi
5.	Bra004982	2	78 261	174 282	ANK ZnF_C3H1
6.	Bra000064	2	316 151	371 205	WRKY WRKY
7.	Bra031073	1	12	522	K_oxygenase superfamily
8.	Bra002283	1	22	122	Stellacyanin
9.	Bra017085	2	21	266	Nodulin-like 2A0111 superfamily
10.	Bra024269	1	36	327	Secretory_peroxidase
11.	Bra023099	1	30	327	Secretory_peroxidase
12.	Bra012806	2	12 185	217 251	Syntaxin SNARE superfamily
13.	Bra033568	1	6	349	UbiH
14.	Bra012938	1	99	157	AP2
15.	Bra000141	1	74	353	STKc_IRAK
16.	Bra022813	1	3	463	p450 superfamily
17.	Bra034754	2	74 21	306 61	Glyco_hydro_19 ChtBD1_GH19_hevein
18.	Bra028635	1	53	294	Phi_1
19.	Bra026986	1	1	450	K_oxygenase superfamily
20.	Bra037520	6	737 620 163 426 239 257	905 835 257 573 285 319	NAD_binding_6 NOX_Duox_like_FAD_NADP NADPH_Ox Ferric_reduct EFh EF-hand_7
21.	Bra000775	1	81	165	HPS_like
22.	Bra021101	1	20	270	lectin_legume_LecRK_Arcelin_ConA
23.	Bra030416	3	34 593 411	353 857 499	Malectin_like STKc_IRAK PLN00113 superfamily
24.	Bra011536	1	57	369	WD40
25.	Bra015272	1	59	155	GlrX-like_plant
26.	Bra037006	2	159 11	430 45	STK_BAK1_like PLN00113 superfamily
27.	Bra034848	2	5 463	441 742	Glycosyltransferase_GTB_type superfamily WD40
28.	Bra003789	1	29	560	PLN02786
29.	Bra022772	1	118	371	PP2Cc
30.	Bra039130	1	10	278	SPFH_like_u4
31.	Bra036316	1	41	272	Chitinase_glyco_hydro_19
32.	Bra028436	1	26	317	Secretory_peroxidase
33.	Bra001422	2	36 209	241 275	Syntaxin SNARE superfamily
34.	Bra029933	1	27	330	Secretory_peroxidase
35.	Bra021184	1	37	252	AUX_IAA
36.	Bra019332	1	1	515	PLN02611
37.	Bra018969	1	38	515	Glyco_hydro_1 superfamily
38.	Bra016675	-	-	-	-
39.	Bra014037	1	21	103	TRX_family
40.	Bra004768	1	28	88	Toxin_3
41.	Bra019407	1	8	119	GABARAP

**Table 3:** Predicted conserved domains in identified up-regulated *Brassica rapa* sequences with their positions under contrast JAMC

S.N.	*B. rapa* accession	No. of conserved domains	From	To	Predicted domain (short name)
1.	Bra006830	1	26	485	p450 superfamily
2.	Bra035148	1	147	205	WRKY
3.	Bra011536	1	57	369	WD40
4.	Bra017561	1	178	235	WRKY
5.	Bra015272	1	59	155	GlrX-like_plant
6.	Bra036260	2	96 4	212 78	GST_C_Phi GST_N_Phi
7.	Bra004982	2	78 261	174 182	ANK ZnF_C3H1
8.	Bra000064	2	316 151	371 205	WRKY WRKY
9.	Bra037006	2	159 11	430 45	STK_BAK1_like PLN00113 superfamily
10.	Bra034848	2	5 463	441 742	Glycosyltransferase_GTB_type superfamily WD40
11.	Bra031073	1	12	522	K_oxygenase superfamily
12.	Bra002283	1	22	122	Stellacyanin
13.	Bra017085	2	21 328	266 483	Nodulin-like 2A0111 superfamily
14.	Bra024269	1	36	327	Secretory_peroxidase
15.	Bra012806	2	12 185	217 251	Syntaxin SNARE superfamily
16.	Bra033568	1	6	349	UbiH
17.	Bra039130	1	10	278	SPFH_like_u4
18.	Bra012938	1	99	157	AP2
19.	Bra000141	1	74	353	STKc_IRAK
20.	Bra022813	1	3	463	p450 superfamily
21.	Bra036316	1	41	272	Chitinase_glyco_hydro_19
22.	Bra026986	1	1	450	K_oxygenase superfamily
23.	Bra028436	1	26	317	Secretory_peroxidase
24.	Bra001422	2	36 209	241 275	Syntaxin SNARE superfamily
25.	Bra000775	1	81	165	HPS_like
26.	Bra021101	1	20	270	Lectin_legume_LecRK_Arcelin_ConA
27.	Bra030416	3	34 593 411	353 857 499	Malectin_like STKc_IRAK PLN00113 superfamily
28.	Bra000754	1	48	312	PKc_like superfamily
29.	Bra003789	1	29	560	PLN02786
30.	Bra022772	1	118	371	PP2Cc
31.	Bra029933	1	27	330	Secretory_peroxidase
32.	Bra019332	1	1	515	PLN02611
33.	Bra011299	1	183	241	WRKY
34.	Bra023099	1	30	327	Secretory_peroxidase
35.	Bra007818	1	38	206	PMT_4TMC superfamily
36.	Bra014037	1	21	103	TRX_family
37.	Bra034754	2	74 21	306 61	Glyco_hydro_19 ChtBD1_GH19_hevein
38.	Bra028635	1	53	294	Phi_1
39.	Bra023609	1	80	335	APG5
40.	Bra014692	1	124	184	WRKY
41.	Bra004768	1	28	88	Toxin_3
42.	Bra019407	1	8	119	GABARAP

**Table 4:** Predicted conserved domains in identified upregulated *Brassica rapa* sequences with their positions under contrast SAMC

S.N.	*B. rapa* accession	No. of conserved domains	From	To	Predicted domain (short name)
1.	Bra006830	1	26	485	p450 superfamily
2.	Bra035148	1	147	205	WRKY
3.	Bra017561	1	178	235	WRKY
4.	Bra036260	2	96 4	212 78	GST_C_Phi GST_N_Phi
5.	Bra004982	2	78 261	174 282	ANK ZnF_C3H1
6.	Bra000064	2	316 151	371 205	WRKY WRKY
7.	Bra002283	1	22	122	Stellacyanin
8.	Bra024269	1	36	327	Secretory_peroxidase
9.	Bra012806	2	12 185	217 251	Syntaxin SNARE superfamily
10.	Bra033568	1	6	349	UbiH
11.	Bra022772	1	118	371	PP2Cc
12.	Bra012938	1	99	157	AP2
13.	Bra000141	1	74	353	STKc_IRAK
14.	Bra022813	1	3	463	p450 superfamily
15.	Bra034754	2	74 21	306 61	Glyco_hydro_19 ChtBD1_GH19_hevein
16.	Bra028635	1	53	294	Phi_1
17.	Bra026986	1	1	450	K_oxygenase superfamily
18.	Bra037520	6	737 620 163 426 239 257	905 835 257 573 285 319	NAD_binding_6 NOX_Duox_like_FAD_NADP NADPH_Ox Ferric_reduct EFh EF-hand_7
19.	Bra028436	1	26	317	Secretory_peroxidase
20.	Bra021101	1	20	270	lectin_legume_LecRK_Arcelin_ConA
21.	Bra029933	1	27	330	Secretory_peroxidase
22.	Bra021184	1	37	252	AUX_IAA
23.	Bra019332	1	1	515	PLN02611
24.	Bra018970	1	133	386	SMC_N superfamily
25.	Bra018969	1	38	515	Glyco_hydro_1 superfamily
26.	Bra023099	1	30	327	Secretory_peroxidase
27.	Bra016675	-	-	-	-
28.	Bra014037	1	21	103	TRX_family
29.	Bra004768	1	28	88	Toxin_3
30.	Bra012551	1	1	463	PLN02196
31	Bra030416	3	34 593 411	353 857 499	Malectin_like STKc_IRAK PLN00113 superfamily
32.	Bra011536	1	57	369	WD40
33.	Bra017656	1	140	202	AP2
34.	Bra037006	2	159 11	430 45	STK_BAK1_like PLN00113 superfamily
35.	Bra005378	3	231 29 375	892 181 464	PLN00113 superfamily PLN00113 superfamily PLN00113 superfamily
36.	Bra039130	1	10	278	SPFH_like_u4
37.	Bra036316	1	41	272	Chitinase_glyco_hydro_19
38.	Bra015454	1	81	412	Abhydrolase superfamily
39.	Bra001422	2	36 209	241 275	Syntaxin SNARE superfamily
40.	Bra000775	1	81	165	HPS_like

**Table 5:** Predicted conserved domains in identified upregulated *Brassica rapa* sequences with their positions under contrast ETMC

S.N.	*B. rapa* accession	No. of conserved domains	From	To	Predicted domain (short name)
1.	Bra006830	1	26	485	p450 superfamily
2.	Bra035148	1	147	205	WRKY
3.	Bra017561	1	178	235	WRKY
4.	Bra015272	1	59	155	GlrX-like_plant
5.	Bra036260	2	96 4	212 78	GST_C_Phi GST_N_Phi
6.	Bra004982	2	78 261	174 282	ANK ZnF_C3H1
7.	Bra000064	2	316 151	371 205	WRKY WRKY
8.	Bra037006	2	159 11	430 45	STK_BAK1_like PLN00113 superfamily
9.	Bra031073	1	12	522	K_oxygenase superfamily
10.	Bra002283	1	22	122	Stellacyanin
11.	Bra017085	2	21 328	266 483	Nodulin-like 2A0111 superfamily
12.	Bra024269	1	36	327	Secretory_peroxidase
13.	Bra012806	2	12 185	217 251	Syntaxin SNARE superfamily
14.	Bra033568	1	6	349	UbiH
15.	Bra022772	1	118	371	PP2Cc
16.	Bra000141	1	74	353	STKc_IRAK
17.	Bra022813	1	3	463	p450 superfamily
18.	Bra028635	1	35	294	Phi_1
19.	Bra026986	1	1	450	K_oxygenase superfamily
20.	Bra037520	6	737 620 163 426 239 257	905 835 257 573 285 319	NAD_binding_6 NOX_Duox_like_FAD_NADP NADPH_Ox Ferric_reduct EFh EF-hand_7
21.	Bra028436	1	26	317	Secretory_peroxidase
22.	Bra004768	1	28	88	Toxin_3
23.	Bra021101	1	20	270	Lectin_legume_LecRK_Arcelin_ConA
24.	Bra029933	1	27	330	Secretory_peroxidase
25.	Bra021184	1	37	252	AUX_IAA
26.	Bra019332	1	1	515	PLN02611
27.	Bra011299	1	183	241	WRKY
28.	Bra018970	1	133	386	SMC_N superfamily
29.	Bra018969	1	38	515	Glyco_hydro_1 superfamily
30.	Bra023099	1	30	327	Secretory_peroxidase
31.	Bra014037	1	21	103	TRX_family
32.	Bra034754	2	74 21	306 61	Glyco_hydro_19 ChtBD1_GH19_hevein
33.	Bra012551	1	1	463	PLN02196
34.	Bra030416	3	34 593 411	353 857 499	Malectin_like STKc_IRAK PLN00113 superfamily
35.	Bra011536	1	57	369	WD40
36.	Bra034848	2	5 463	441 742	Glycosyltransferase_GTB_type superfamily WD40
37.	Bra003789	1	29	560	PLN02786
38.	Bra039130	1	10	278	SPFH_like_u4
39.	Bra012938	1	99	157	AP2
40.	Bra036316	1	41	272	chitinase_glyco_hydro_19
41.	Bra001422	2	36 209	241 275	Syntaxin SNARE superfamily
42.	Bra000775	1	81	165	HPS_like

### Topological analysis and visualization of PPI network for identification of key components involved through JA-SA-ET-mediated resistance

After analysis, the identified upregulated genes of *Arabidopsis thaliana* involved in defense response to fungal pathogen at 9 and 24 h for the contrast JAMC, SAMC, and ETMC were chosen to build extended PPI network based on confidence score > 0.7 as cutoff. The constructed networks were visualized and analyzed by Cytoscape 3.4.0 and Network Analyzer 3.3.1. Network analysis revealed that the JAMC network has 34 nodes, 68 edges, 3 connected components, 0 isolated node, 4.0 average number of neighbors, 600 shortest paths, 2.807 characteristic path lengths, 7 network diameters, and 1 network radius; SAMC network has 33 nodes, 56 edges, 5 connected components, 0 isolated node, 3.394 average number of neighbors, 380 shortest paths, 2.053 characteristic path lengths, 4 network diameters, and 1 network radius; ETMC network has 36 nodes, 57 edges, 6 connected components, 0 isolated node, 3.167 average number of neighbors, 420 shortest paths, 2.167 characteristic path lengths, 5 network diameters, and 1 network radius (Table 6).

**Table 6:** Values of topological parameters

Parameters	JAMC	SAMC	ETMC
Node	34	33	36
Edge	68	56	57
CC	3	5	6
ANN	4.0	3.394	3.167
SP	600	380	420
CPL	2.807	2.053	2.167
ND	7	4	5
MENP	0	0	0
IN	0	0	0
NR	1	1	1

*CC* connected component, *ANN* average number of neighbors, *SP* shortest path, *CPL* characteristics path length, *ND* network diameter, *MENP* multi-edge node pair, *IN* isolated node, *NR* network radius

**Fig. 7:**
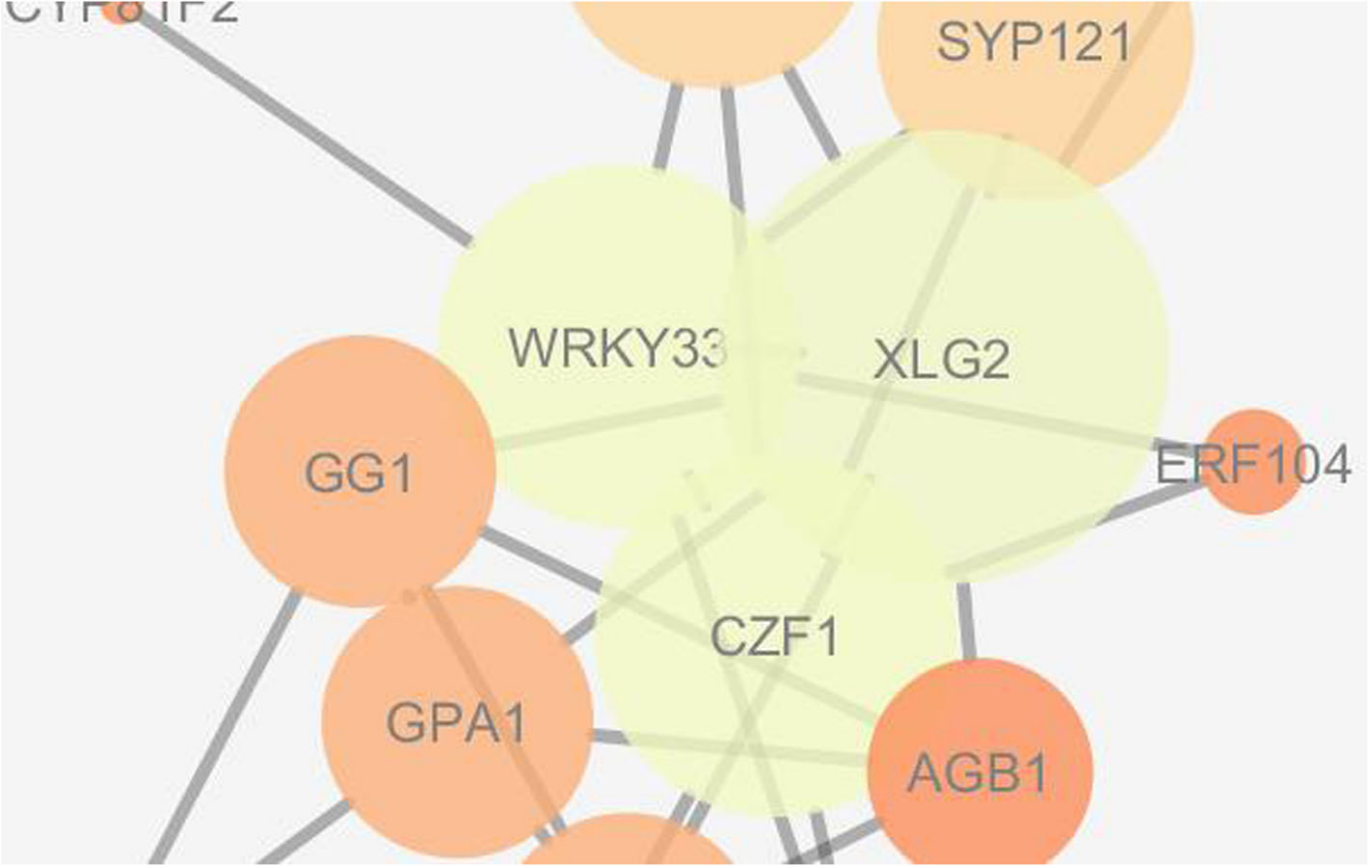
Visualization of key components involved in JA-mediated resistance in *Arabidopsis thaliana* under contrast JAMC

**Fig. 8:**
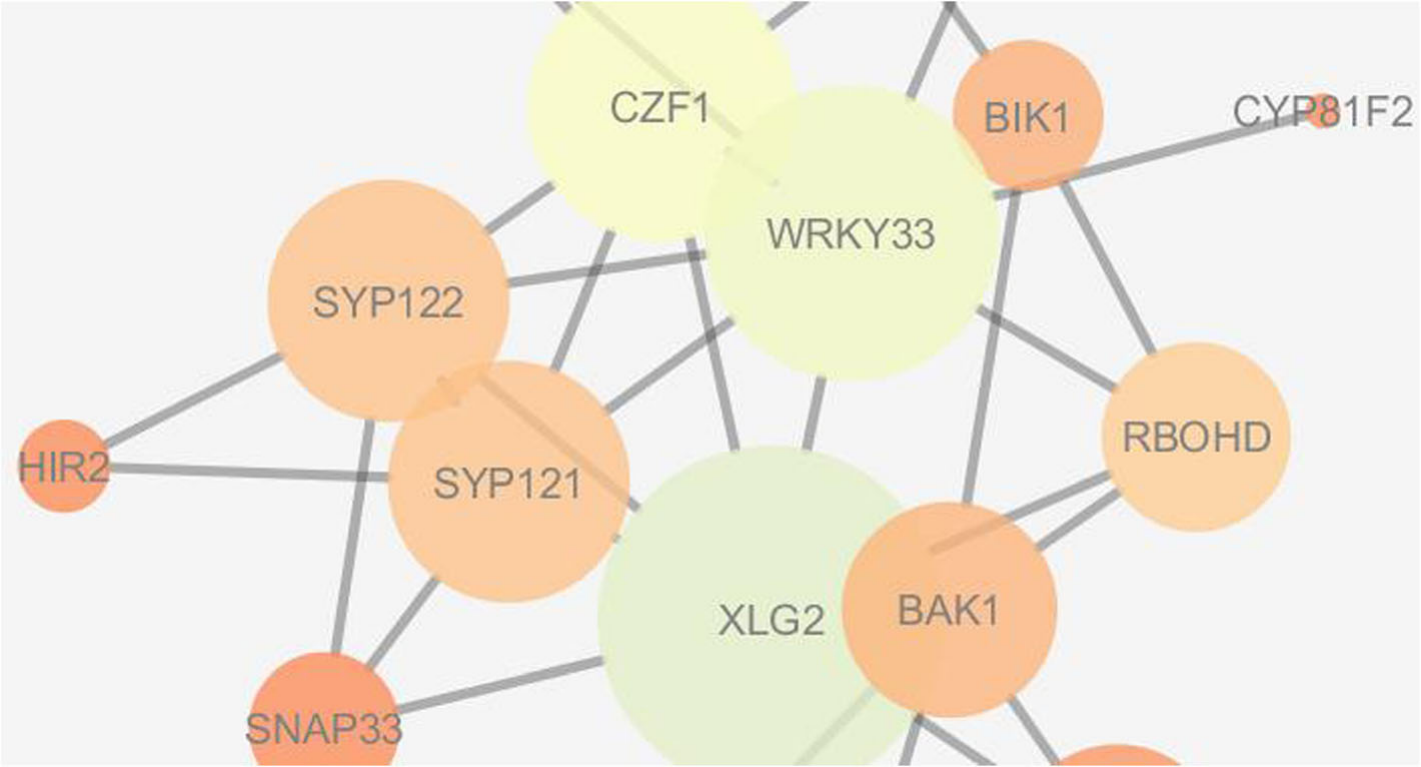
Visualization of key components involved in SA-mediated resistance in *Arabidopsis thaliana* under contrast SAMC

**Fig. 9:**
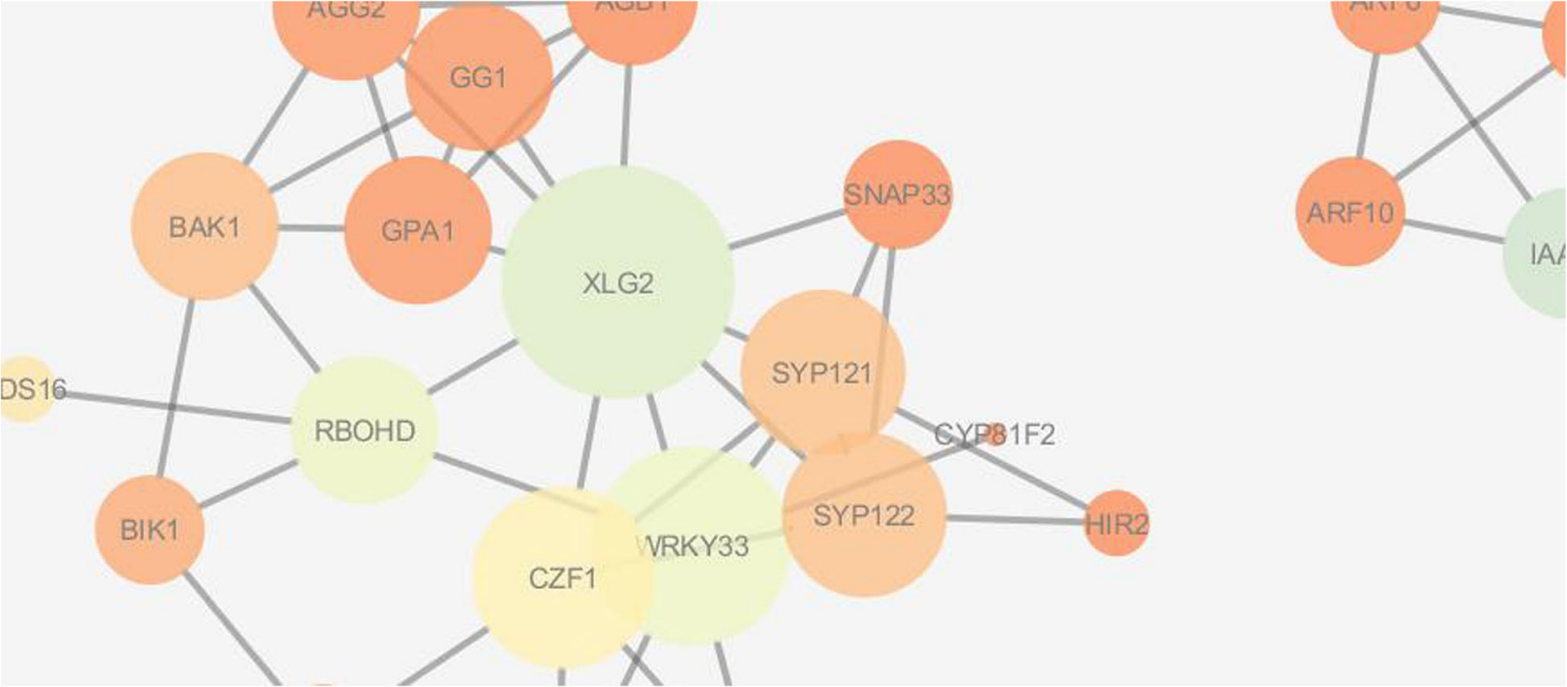
Visualization of key components involved in *ET*-mediated resistance in *Arabidopsis thaliana* under contrast ETMC

The visual parameter of NetworkAnalyzer was used to map hub nodes in the networks using the visual style to map node size “Degree” and node color “BetweenessCentrality” to investigate the key components of *JA-SA-ET-*mediated pathway triggered during resistance. The nodes MP, IAA19, AXR3, IAA1, ARF6, and XLG2 were found as significant components and XLG2, WRKY33, and CZF1 are found as hub nodes under contrast JAMC, SAMC, and ETMC, which play tremendous role during plant-pathogen interaction (Figs. 7, 8, 9).

## Discussion

In the present study, efforts have been made to identify and characterize resistant and defense-related genes triggered during resistance towards *Alternaria* blight, a recalcitrant disease caused by *Alternaria brassicicola* and *Alternaria brassicae* in *Arabidopsis* and *Brassica*. Besides, the key components of JA-SA-ET involved in defense response were also investigated. The identified top ten resistant and defense-related genes are listed in Tables S2-S17. These genes could be utilized for development of molecular markers linked with disease resistance which can further be utilized in molecular breeding program. Moreover, the results can also be utilized for transgenesis, directed mutagenesis, cisgenesis, and gene editing for development of resistant *Brassica* plants against *Alternaria* blight.

The identification of resistance (R) and defense-related genes unlocked interesting possibilities for prevention and management of diseases caused by several pathogens [42]. However, such genes are available in limited numbers which can be deployed in plants to engineer defense against limited number of pathogens. On the other hand, efficient application of microarray technology and functional genomics tools allow us to discover important candidate genes through stimulating better understanding of disease resistance and plant defense signaling. It could disclose novel insights on the interactions among signaling pathways and other processes of plant systems involved in plant-pathogen interactions [43, 44]. Various studies as conducted in recent past on the signaling machinery towards necrotrophic fungal pathogens have helped to dissect various components.

The knowledge on molecular mechanism of host pathogen interaction is considered to be prerequisite for engineering disease-resistant varieties of *Brassica* against *Alternaria* blight disease. Throughout plant-pathogen interactions, our knowledge of responses has taken a big leap forward. Nonetheless, over the course of this decade, we still have several aspects and challenges to address different questions associated to these interactions [45]. It is believed that the huge data on expression of resistance and defense related genes with respect to plant-pathogen interaction can be analyzed to identify key candidate gene(s) which can be modified by genetic engineering or molecular breeding approaches to engineer disease resistance in *Brassica*.

Recently efforts have been made in dissecting the different components of defense signal transduction pathways activated towards different pathogens. Jasmonic acid/ethylene and salicylic acid-mediated signaling pathways are activated against necrotrophic and biotrophic fungal pathogens [7, 46]. *Arabidopsis thaliana* has already been demonstrated as host for *Alternaria* blight disease of *Brassica* [47]. Therefore, it is being felt that *Alternaria brassicicola-Arabidopsis thaliana* could be used as one of the excellent model system for deciphering the intricacy of *Alternaria* blight in *Brassica* [4]. Pre-processing of microarray data is the phenomenon of extracting and transforming the intensities of raw fluorescence into a signal normalized for biological variations and experimental errors [48]. Here, GCRMA (Guanine Cytosine Robust Multi-Array Analysis) method was used for background correction of downloaded microarray data from NCBI GEO [49]. It converts background adjusted probe intensities into expression measures as same has been used by RMA (Robust Multi-array Average) for normalization and summarization of data [49]. It performs much better than the other commonly used methods for normalization [27] to identify upregulated and downregulated DEGs*.* The present study has demonstrated different sets of differentially expressed JA, SA, and ET responsive genes (DEGs) at 9 and 24 h after infection of *Alternaria brasscicola.* All the upregulated and downregulated DEGs given in parenthesis for each contrast, i.e., wild-type pathogen-treated plant with control plant (WT9C9: 1327up, 1527down; WT24C24: 1510up, 1455down); jasmonic acid mutant pathogen-treated plant with control (JAM9C9: 809up, 350down; JAM24C24: 2201up, 2397down); salicylic acid mutant pathogen-treated plant with control (SAM9C9: 1355up, 1161down; SAM24C24: 1819up, 1984down); ethylene mutant-treated plant with control (ETM9C9: 917up, 650down; ETM24C24: 1895up, 2269down) at 9 h and 24 h after *Alternaria brassicicola* infection. During data analysis and annotation, among many defense related genes, NHL10 and HCHIB were recognized as resistance genes. The NHL10 is non-race-specific disease resistance gene (NDR1) [50], and HCHIB is involved in ethylene/jasmonate-mediated systemic acquired resistance during pathogenesis. They are involved in defense responses during pathogenesis of *Alternaria* blight in *Arabidopsis thaliana* [51, 52]. Besides, enrichment analyses of the DEGs led to formation of gene ontology to define the significant genes involved in biological processes, molecular function, cellular components, and pathways. The genes involved in defense response towards fungi in *Arabidopsis thaliana* were mapped on *Brassica rapa* genome sequences to identify and characterize the similar *Brassica* sequences. Characterization and comparative analysis of identified genes were carried through molecular phylogeny analysis and domain prediction to identify resistance and defense-related genes in *Brassica*. Infection of *Alternaria brasscicola* led to upregulation of various genes such as WRKY, peroxidase, p450 oxidases, and chitinase which mediate defense response in *Arabidopsis* and *Brassica* upon infection with pathogen [53–56]. It has been observed that the expression of these genes increase more in the presence of JA and SA than the wild-type plants at 24 h post infection. This indicates that expression of defense-related genes increase post infection of *Alternaria brasscicola* to combat pathogen’s spread and this effect is enhanced by JA and SA which are well-known defense inducers. However, there were observed few genes which were downregulated in the presence of JA and SA. These genes appear to be the ones which are involved in pathogenesis process and that they are downregulated by JA or SA to trigger defense response against the pathogen. Protein-protein interaction networks of genes involved in defense response towards fungi were constructed from contrast JAMC, SAMC, and ETMC to determine the key components of *JA-SA-ET*-mediated pathway involved in disease resistance through network analysis. The genes, *CZF1*; *WRKY*; Movement Protein (MP); INDOLE-3-ACETIC ACID INDUCIBLE 19 (IAA19); Auxin-responsive gene (AXR3); INDOLE-3-ACETIC ACID INDUCIBLE 1 (IAA1); auxin response factor 6 (ARF6); and extra large G-protein 2 (XLG2) involved in DNA-binding transcription factor activity, GTP binding, GTPase activity, and protein binding as well as defense response towards fungi were investigated [55]. However, XLG2 which is found in all contrast in the category of hubs is a well-characterized gene playing significant role in disease resistance [57].

## Conclusion

In the present computational study, among many defense-related genes, NHL10 and HCHIB were identified as major genes which are involved in defense responses during pathogenesis of *Alternaria* blight in *Arabidopsis thaliana*. Besides, the key components of the three main signaling pathway, viz., jasmonic acid, salicylic acid, and ethylene-mediated pathway triggered during resistance were also identified. The genes, viz., *CZF1*, *WRKY*, MP, IAA19, AXR3, IAA1, ARF6, and XLG2 were found as potential candidate genes of these signaling pathways. Additionally, XLG2 was found to be one of the most promising key genes involved in defense response against *Alternaria brasscicola* fungal pathogen. Furthermore, the genes involved in defense response to *Alternaria brasscicola* were also identified and characterized in *Brassica rapa* by taking *Arabidopsis* as a model system. The finding from the present study may provide a way to understand the intricate molecular mechanism of *Brassica-Alternaria* pathosystem. This may further be used for devising strategies based on molecular breeding or genetic engineering approaches to develop designer resistant *Brassica* crops for robust oilseed productivity and sustainability, and securing food and nutritional security of rapidly growing world population.

## Supplementary information

**Additional file 1.** Supplementary Tables

## Data Availability

Not applicable

## Abbreviations

NCBI: National Center for Biotechnology Information
GEO: Gene Expression Omnibus
BLAST: Basic Local Alignment Search Tool
GCRMA: Guanine Cytosine Robust Multi-Array Analysis
JA: Jasmonic acid
SA: Salicylic acid
ET: Ethylene
WTC: Wild-type pathogen-treated plant vs control plant
JAMC: Jasmonic acid mutant with pathogen-treated plant vs control plant
SAMC: Salicylic acid mutant with pathogen-treated plant vs control plant
ETMC: Ethylene mutant with pathogen-treated plant vs control plant
DEGs: Differentially expressed genes
TAIR: The Arabidopsis Information Resource
CC: Connected component
ANN: Average number of neighbors
SP: Shortest path
CPL: Characteristics path length
ND: Network diameter
MENP: Multi-edge node pair
IN: Isolated node
NR: Network radius
