# Supplementary material for: Computational analysis of microarray data of Arabidopsis thaliana challenged with Alternaria brassicicola for identification of key genes in Brassica
Source: J Genet Eng Biotechnol. 2020 Jul 1;18:17. doi: 10.1186/s43141-020-00032-y (PMC7326868; doi:10.1186/s43141-020-00032-y)
Supplement: Supplementary file 1 — Additional file 1. Supplementary Tables [file 43141_2020_32_MOESM1_ESM.docx]

**Supplementary Table 1. Top ten upregulated genes (WT9C9).**

| **S.N.** | **ProbeID** | **Fold Change** | **P-value** | **EntrezID** | **TAIR Accession** | **Gene Symbol** | **Description** |
| --- | --- | --- | --- | --- | --- | --- | --- |
| 1 | 263403_at | 7.363658229 | 1.39E-14 | 814938 | AT2G04040 | ATDTX1 | It has been investigated as a detoxifying efflux carrier for antibiotics and other compounds derived from plants |
| 2 | 254818_at | 8.218638578 | 5.90E-13 | 826859 | AT4G12470 | AZI1 | It is involved in systemic immunity triggered by pathogen |
| 3 | 248686_at | 4.200091445 | 5.91E-13 | 834910 | AT5G48540 | NA | Receptor-like protein kinase-related family protein |
| 4 | 263948_at | 8.366925414 | 1.15E-12 | 818171 | AT2G35980 | ATNHL10 | Arabidopsis non-race specific disease resistance gene (NDR1) |
| 5 | 251745_at | 3.290217302 | 1.21E-12 | 824764 | AT3G55980 | ATSZF1 | Salt-inducible zinc finger 1 |
| 6 | 253416_at | 5.536134994 | 1.47E-12 | 829444 | AT4G33070 | AtPDC1 | Thiamine pyrophosphate dependent pyruvate decarboxylase protein family |
| 7 | 259979_at | 4.314411336 | 1.78E-12 | 843993 | AT1G76600 | NA | Poly polymerase |
| 8 | 265725_at | 5.249198731 | 2.70E-12 | 817763 | AT2G32030 | NA | Acyl-CoA N-acyltransferases (NAT) superfamily protein. |
| 9 | 245317_at | 6.212631089 | 2.72E-12 | 827238 | AT4G15610 | NA | Uncharacterized protein |
| 10 | 252214_at | 4.01307321 | 6.39E-12 | 824188 | AT3G50260 | ATERF#011 | Involved in defense and freezing stress responses. |

**Supplementary Table 2. Top ten upregulated genes (WT24C24).**

| **S.N.** | **ProbeID** | **Fold Change** | **P-value** | **EntrezID** | **TAIR Accession** | **Gene Symbol** | **Description** |
| --- | --- | --- | --- | --- | --- | --- | --- |
| 1 | 258791_at | 5.748666881 | 2.40E-14 | 819632 | AT3G04720 | AtPR4 | It encodes a protein like the anti-fungal chitin-binding protein. Its expression is increases in response to ethylene and turnip crinkle virus infection. |
| 2 | 256243_at | 7.964273388 | 3.40E-14 | 820429 | AT3G12500 | ATHCHIB | It is involved in ethylene/ jasmonate mediated systemic acquired resistance during pathogenesis. |
| 3 | 245628_at | 3.02728725 | 5.81E-14 | 842120 | AT1G56650 | ATMYB75 | Interacts with JAZ proteins to regulate anthocyanin accumulation. |
| 4 | 248138_at | 4.412779173 | 1.36E-13 | 835587 | AT5G54960 | PDC2 | Pyruvate decarboxylase-2 |
| 5 | 262482_at | 3.915768331 | 2.95E-13 | 838272 | AT1G17020 | ATSRG1 | Senescence-related gene |
| 6 | 249052_at | 8.960886431 | 7.70E-13 | 834469 | AT5G44420 | LCR77 | Encodes an jasmonate and ethylene responsive plant defensin. |
| 7 | 254889_at | 10.02170521 | 9.57E-13 | 826770 | AT4G11650 | ATOSM34 | Osmotin-like protein |
| 8 | 245317_at | 7.188530841 | 1.46E-12 | 827238 | AT4G15610 | NA | Uncharacterized protein family |
| 9 | 258957_at | 6.781758859 | 1.92E-12 | 821135 | AT3G01420 | ALPHA-DOX1 | It encodes an alpha-dioxygenase which is involved in defense against oxidative stress and cell death. It is induced in response to oxidative stress and salicylic acid |
| 10 | 257365_x_at | 8.175752611 | 2.50E-12 | 817143 | AT2G26020 | PDF1.2b | It is belongs to defensin family and predicted to encode a PR (Pathogenesis related protein) |

**Supplementary Table 3. Top ten downregulated genes (WT9C9).**

| **S.N.** | **ProbeID** | **Fold Change** | **P-value** | **EntrezID** | **TAIR Accession** | **Gene Symbol** | **Description** |
| --- | --- | --- | --- | --- | --- | --- | --- |
| 1 | 255041_at | -1.334752993 | 2.94E-09 | 826547 | AT4G09620 | NA | Mitochondrial transcription termination factor family protein. |
| 2 | 245797_at | -1.54560566 | 9.45E-09 | 841096 | AT1G45230 | NA | DCL protein |
| 3 | 257827_at | -1.512894012 | 1.41E-08 | 822275 | AT3G26630 | NA | Tetratricopeptide repeat (TPR)-like superfamily protein |
| 4 | 252243_at | -1.196741211 | 2.58E-08 | 824174 | AT3G50120 | NA | Transmembrane protein |
| 5 | 265967_at | -1.781863181 | 3.47E-08 | 818322 | AT2G37450 | UMAMIT13 | Nodulin MtN21-like transporter family protein |
| 6 | 266951_at | -2.26421417 | 3.73E-08 | 816411 | AT2G18940 | NA | Tetratricopeptide repeat (TPR)-like superfamily protein |
| 7 | 249129_at | -1.235223761 | 6.07E-08 | 834324 | AT5G43080 | CYCA3;1 | Cyclin A3 |
| 8 | 264963_at | -1.834423949 | 8.63E-08 | 842354 | AT1G60600 | ABC4 | It is involved in plastoquinone and phylloquinone biosynthesis. |
| 9 | 261519_at | -1.497138342 | 1.31E-07 | 843511 | AT1G71810 | NA | Protein kinase superfamily protein |
| 10 | 257831_at | -1.313283921 | 1.60E-07 | 822283 | AT3G26710 | CCB1 | Cofactor assembly of complex C |

**Supplementary Table 4. Top ten downregulated genes (WT24C24).**

| **S.N.** | **ProbeID** | **Fold Change** | **P-value** | **EntrezID** | **TAIR Accession** | **Gene Symbol** | **Description** |
| --- | --- | --- | --- | --- | --- | --- | --- |
| 1 | 248423_at | -1.207098216 | 2.74E-27 | 835241 | AT5G51670 | NA | Hypothetical protein |
| 2 | 259417_at | -0.205619585 | 6.01E-17 | 839300 | AT1G02340 | FBI1 | It is involved in phytochrome signaling |
| 3 | 261658_at | -1.720231004 | 8.37E-13 | 841428 | AT1G50040 | NA | Formin-like protein, putative |
| 4 | 263480_at | -1.743553587 | 1.73E-12 | 814931 | AT2G04032 | ZIP7 | Zinc transporter 7 precursor |
| 5 | 262980_at | -3.399122604 | 2.18E-11 | 843902 | AT1G75680 | AtGH9B7 | Glycosyl hydrolase 9B7 |
| 6 | 264978_at | -3.464563955 | 9.10E-11 | 839601 | AT1G27120 | NA | Encodes a Golgi-localized hydroxyproline-O-galactosyltransferase. |
| 7 | 262414_at | -2.490453291 | 1.81E-10 | 841367 | AT1G49430 | LACS2 | It encodes a long chain acyl-CoA synthetase that is involved in the catalysis of Omega-hydroxy fatty acyl-CoA intermediates synthesis in the pathway of cutin synthesis |
| 8 | 264371_at | -4.62544359 | 3.79E-10 | 837761 | AT1G12090 | ELP | Extensin-like protein |
| 9 | 253254_at | -1.250609511 | 6.93E-10 | 829617 | AT4G34650 | SQS2 | It is involved in sterol biosynthesis |
| 10 | 247881_at | -2.811771378 | 7.49E-10 | 835877 | AT5G57700 | NA | BNR/Asp-box repeat family protein. |

**Supplementary Table 5. Top ten upregulated genes (JAM9C9).**

| **S.N.** | **ProbeID** | **Fold Change** | **P-value** | **EntrezID** | **TAIR Accession** | **Gene Symbol** | **Description** |
| --- | --- | --- | --- | --- | --- | --- | --- |
| 1 | 263403_at | 6.828181122 | 5.67E-14 | 814938 | AT2G04040 | ATDTX1 | It has been investigated as a detoxifying efflux carrier for antibiotics and other compounds derived from plants |
| 2 | 251745_at | 3.855237698 | 6.62E-14 | 824764 | AT3G55980 | ATSZF1 | Salt-inducible zinc finger 1 |
| 3 | 253416_at | 6.228211889 | 1.70E-13 | 829444 | AT4G33070 | AtPDC1 | Thiamine pyrophosphate dependent pyruvate decarboxylase family protein. |
| 4 | 254818_at | 8.334741984 | 4.57E-13 | 826859 | AT4G12470 | AZI1 | It is involved in systemic immunity triggered by pathogen |
| 5 | 261450_s_at | 6.687395408 | 5.55E-13 | 838707 | AT1G21110 | IGMT3 | O-methyltransferase family protein. |
| 6 | 248686_at | 4.079570472 | 1.01E-12 | 834910 | AT5G48540 | NA | Receptor-like protein kinase-related family protein. |
| 7 | 250090_at | 6.300423889 | 2.90E-12 | 831599 | AT5G17330 | GAD | Encodes one of two isoforms of glutamate decarboxylase. |
| 8 | 265725_at | 5.222531153 | 2.96E-12 | 817763 | AT2G32030 | NA | Acyl-CoA N-acyltransferases (NAT) superfamily protein. |
| 9 | 263948_at | 7.688590226 | 5.29E-12 | 818171 | AT2G35980 | ATNHL10 | Arabidopsis non-race specific disease resistance gene (NDR1) |
| 10 | 254543_at | 4.482074598 | 7.21E-12 | 827725 | AT4G19810 | ChiC | Its transcript level rise in response to fungal and bacterial pathogens. |

**Supplementary Table 6. Top ten upregulated genes (JAM24C24).**

| **S.N.** | **ProbeID** | **Fold Change** | **P-value** | **EntrezID** | **TAIR Accession** | **Gene Symbol** | **Description** |
| --- | --- | --- | --- | --- | --- | --- | --- |
| 1 | 248887_at | 0.540347209 | 5.43E-21 | 834653 | AT5G46115 | NA | Hypothetical protein |
| 2 | 256190_at | 6.376900756 | 3.62E-15 | 839889 | AT1G30100 | ATNCED5 | It involved in the biosynthesis of abscisic acid |
| 3 | 262482_at | 4.148357795 | 1.28E-14 | 838272 | AT1G17020 | ATSRG1 | Senescence-related gene |
| 4 | 265720_at | 3.43534662 | 2.13E-14 | 818600 | AT2G40110 | NA | Yippee family putative zinc-binding protein. |
| 5 | 245317_at | 7.467488917 | 9.40E-14 | 827238 | AT4G15610 | NA | Uncharacterized protein family. |
| 6 | 253454_at | 0.830522564 | 1.28E-13 | 829317 | AT4G31875 | NA | Hypothetical protein. |
| 7 | 250648_at | 1.413868104 | 1.90E-13 | 830565 | AT5G06760 | AtLEA4-5 | Accumulates in response to low water availability during growth and development |
| 8 | 267280_at | 2.164516023 | 3.80E-13 | 816464 | AT2G19450 | ABX45 | Involved in senescence and seed development |
| 9 | 245158_at | 2.442599005 | 6.47E-13 | 817875 | AT2G33130 | RALF18 | Similar to tobacco rapid alkalization factor (RALF). Play essential role in physiology |
| 10 | 258957_at | 6.165088686 | 1.43E-12 | 821135 | AT3G01420 | ALPHA-DOX1 | It encodes an alpha-dioxygenase which is involved in defense against oxidative stress and cell death. It is induced in response to oxidative stress and salicylic acid |

**Supplementary Table 7. Top ten downregulated genes (JAM9C9).**

| **S.N.** | **ProbeID** | **Fold Change** | **P-value** | **EntrezID** | **TAIR Accession** | **Gene Symbol** | **Description** |
| --- | --- | --- | --- | --- | --- | --- | --- |
| 1 | 249208_at | -4.85527684 | 6.38E-14 | 834273 | AT5G42650 | AOS | It is involved in the JA biosynthesis pathway to catalyze the dehydration of the hydroperoxide to an unstable allene oxide. |
| 2 | 265118_at | -2.353404869 | 1.77E-07 | 842563 | AT1G62660 | NA | Glycosyl hydrolases family 32 protein. |
| 3 | 261588_at | -0.438931921 | 2.73E-07 | 839365 | AT1G01670 | NA | RING/U-box superfamily protein |
| 4 | 256577_at | -4.265543869 | 3.60E-07 | 822448 | AT3G28220 | NA | TRAF-like family protein |
| 5 | 260205_at | -2.90734701 | 7.12E-07 | 843407 | AT1G70700 | JAZ9 | It is an essential protein of JA signalling. It interacts with JA receptor COI1 F-box subunit of an SCF E3 ubiquitin ligase. |
| 6 | 249052_at | -3.404761239 | 1.12E-06 | 834469 | AT5G44420 | LCR77 | Encodes an jasmonate and ethylene responsive plant defensin. |
| 7 | 259640_at | -6.272569826 | 1.16E-06 | 841670 | AT1G52400 | ATBG1 | Found in ER body, it is required in inducible ER body formation. |
| 8 | 251485_at | -0.469981559 | 1.48E-06 | 825124 | AT3G59550 | ATRAD21.2 | It is essential for megagametogenesis and plays a vital role in pollen development. |
| 9 | 251990_at | -0.692273239 | 1.93E-06 | 824500 | AT3G53320 | NA | Encodes a microtubule-associated protein |
| 10 | 260224_at | -0.56261982 | 2.12E-06 | 843781 | AT1G74400 | NA | Tetratricopeptide repeat (TPR)-like superfamily protein. |

**Supplementary Table 8. Top ten downregulated genes (JAM24C24).**

| **S.N.** | **ProbeID** | **Fold Change** | **P-value** | **EntrezID** | **TAIR Accession** | **Gene Symbol** | **Description** |
| --- | --- | --- | --- | --- | --- | --- | --- |
| 1 | 248423_at | -1.227294864 | 2.26E-28 | 835241 | AT5G51670 | NA | Hypothetical protein |
| 2 | 259417_at | -0.223197236 | 1.49E-18 | 839300 | AT1G02340 | FBI1 | It is involved in phytochrome signaling |
| 3 | 263480_at | -1.75961261 | 1.91E-13 | 814931 | AT2G04032 | ZIP7 | Zinc transporter 7 precursor |
| 4 | 261658_at | -1.63090423 | 2.88E-13 | 841428 | AT1G50040 | NA | Formin-like protein |
| 5 | 249208_at | -3.584557153 | 1.59E-11 | 834273 | AT5G42650 | AOS | It is involved in the JA biosynthesis pathway to catalyze the dehydration of the hydroperoxide to an unstable allene oxide. |
| 6 | 262309_at | -4.08372869 | 4.38E-11 | 843419 | AT1G70820 | NA | Phosphoglucomutase, putative / glucose phosphomutase |
| 7 | 253254_at | -1.250432004 | 1.02E-10 | 829617 | AT4G34650 | SQS2 | It catalyzes the first committed step in sterol biosynthesis |
| 8 | 250413_at | -2.347771622 | 1.53E-10 | 830985 | AT5G11160 | APT5 | Adenine phosphoribosyltransferase 5 |
| 9 | 262414_at | -2.247764748 | 1.55E-10 | 841367 | AT1G49430 | LACS2 | It encodes a long chain acyl-CoA synthetase that is involved in the catalysis of Omega-hydroxy fatty acyl-CoA intermediates synthesis in the pathway of cutin synthesis |
| 10 | 255447_at | -2.640883411 | 2.23E-10 | 828174 | AT4G02790 | EMB3129 | It encodes a GTPase that is targeted to chloroplasts. |

**Supplementary Table 9. Top ten upregulated genes (SAM9C9).**

| **S.N.** | **ProbeID** | **Fold Change** | **P-value** | **EntrezID** | **TAIR Accession** | **Gene Symbol** | **Description** |
| --- | --- | --- | --- | --- | --- | --- | --- |
| 1 | 251745_at | 4.53899509 | 3.14E-15 | 824764 | AT3G55980 | ATSZF1 | Salt-inducible zinc finger 1 |
| 2 | 263403_at | 7.814390275 | 4.57E-15 | 814938 | AT2G04040 | ATDTX1 | It has been investigated as a detoxifying efflux carrier for antibiotics and other compounds derived from plants |
| 3 | 253416_at | 6.477190941 | 8.26E-14 | 829444 | AT4G33070 | AtPDC1 | Thiamine pyrophosphate dependent pyruvate decarboxylase protein family |
| 4 | 265725_at | 6.156897528 | 1.47E-13 | 817763 | AT2G32030 | NA | Acyl-CoA N-acyltransferases (NAT) superfamily protein. |
| 5 | 261450_s_at | 7.170519412 | 1.54E-13 | 838707 | AT1G21110 | IGMT3 | O-methyltransferase family protein |
| 6 | 259979_at | 4.885458323 | 1.84E-13 | 843993 | AT1G76600 | NA | Poly polymerase |
| 7 | 257918_at | 6.968846002 | 3.43E-13 | 821901 | AT3G23230 | AtERF98 | Encodes a member of the ethylene response factor (ERF) subfamily B-3 of ERF/AP2 TF family. |
| 8 | 254818_at | 8.23316996 | 5.71E-13 | 826859 | AT4G12470 | AZI1 | It is involved in systemic immunity triggered by pathogen |
| 9 | 248686_at | 4.170530164 | 6.73E-13 | 834910 | AT5G48540 | NA | Receptor-like protein kinase-related family protein. |
| 10 | 263948_at | 8.554208783 | 7.66E-13 | 818171 | AT2G35980 | ATNHL10 | Arabidopsis non-race specific disease resistance gene (NDR1) |

**Supplementary Table 10. Top ten upregulated genes (SAM24C24).**

| **S.N.** | **ProbeID** | **Fold Change** | **P-value** | **EntrezID** | **TAIR Accession** | **Gene Symbol** | **Description** |
| --- | --- | --- | --- | --- | --- | --- | --- |
| 1 | 245628_at | 4.838480179 | 9.86E-19 | 842120 | AT1G56650 | ATMYB75 | It is a transcription factor interacts with JAZ proteins to regulate anthocyanin accumulation. |
| 2 | 256589_at | 3.943916953 | 1.16E-15 | 822506 | AT3G28740 | CYP81D11 | Its over-expression leads to the synthesis of volatile compounds that affect insect interactions and chemical ecology. |
| 3 | 248138_at | 5.020801964 | 1.53E-15 | 835587 | AT5G54960 | PDC2 | Pyruvate decarboxylase-2 |
| 4 | 258791_at | 5.789899874 | 2.61E-15 | 819632 | AT3G04720 | AtPR4 | It encodes a protein like the anti-fungal chitin-binding protein. Its expression is increases in response to ethylene and turnip crinkle virus infection. |
| 5 | 262482_at | 4.482117635 | 3.01E-15 | 838272 | AT1G17020 | ATSRG1 | Senescence-related gene |
| 6 | 260563_at | 3.312783648 | 7.12E-15 | 818988 | AT2G43840 | UGT74F1 | The true biological substrate(s) of UGT74F1 are not identified yet, but the mutant plants lacking UGT74F1 hold a decreased salicylate glucoside level |
| 7 | 256243_at | 7.549748233 | 1.15E-14 | 820429 | AT3G12500 | ATHCHIB | It is involved in ethylene/ jasmonate mediated systemic acquired resistance during pathogenesis. |
| 8 | 246042_at | 3.226761566 | 5.89E-14 | 832064 | AT5G19440 | NA | Similar to Eucalyptus gunnii alcohol dehydrogenase of unidentified physiological function |
| 9 | 249052_at | 9.094894421 | 7.54E-14 | 834469 | AT5G44420 | LCR77 | Encodes an jasmonate and ethylene responsive plant defensin. |
| 10 | 260783_at | 6.666512524 | 1.05E-13 | 837125 | AT1G06160 | ORA59 | It encodes a member of the ethylene response factor (ERF) subfamily B-3 of ERF/AP2 TF family |

**Supplementary Table 11. Top ten downregulated genes (SAM9C9).**

| **S.N.** | **ProbeID** | **Fold Change** | **P-value** | **EntrezID** | **TAIR Accession** | **Gene Symbol** | **Description** |
| --- | --- | --- | --- | --- | --- | --- | --- |
| 1 | 256617_at | -1.367201069 | 1.92E-11 | 821794 | AT3G22240 | NA | Cysteine-rich/transmembrane domain PCC1-like protein |
| 2 | 248169_at | -5.253887573 | 6.98E-11 | 835550 | AT5G54610 | ANK | Induced in response to Salicylic acid. Belongs to the ankyrin repeat protein family. |
| 3 | 265837_at | -4.627630817 | 7.13E-11 | 815943 | AT2G14560 | LURP1 | Upregulated after infection of pathogenic oomycte *Hyaloperonospora parasitica.* It required for defense responses and resistance to this pathogen which is mediated by R proteins RPP4 and RPP5. |
| 4 | 266385_at | -5.875550361 | 3.04E-10 | 815949 | AT2G14610 | ATPR1 | PR1 gene expression is induced in response to a number of pathogens. It is utilized as molecular marker for the SAR response. |
| 5 | 265611_at | -1.086020628 | 2.10E-09 | 817089 | AT2G25510 | NA | Transmembrane protein |
| 6 | 256766_at | -2.396146783 | 3.26E-09 | 821788 | AT3G22231 | PCC1 | Upregulated in response to virulent and avirulent strains of *P. syringae* pv. tomato |
| 7 | 250942_at | -5.477505701 | 6.17E-09 | 831863 | AT5G03350 | NA | Legume lectin family protein. |
| 8 | 262374_s_at | -1.507164058 | 6.63E-09 | 843622 | AT1G72910 | NA | Toll-Interleukin-Resistance (TIR) domain-containing protein. |
| 9 | 250689_at | -1.655408007 | 2.10E-08 | 830549 | AT5G06610 | NA | Lipase |
| 10 | 247684_at | -5.447667547 | 2.89E-08 | 836088 | AT5G59670 | NA | Leucine-rich repeat protein kinase family protein. |

**Supplementary Table 12. Top ten downregulated genes (SAM24C24).**

| **S.N.** | **ProbeID** | **Fold Change** | **P-value** | **EntrezID** | **TAIR Accession** | **Gene Symbol** | **Description** |
| --- | --- | --- | --- | --- | --- | --- | --- |
| 1 | 248423_at | -1.235456201 | 1.99E-28 | 835241 | AT5G51670 | NA | Hypothetical protein |
| 2 | 259417_at | -0.223197236 | 1.49E-18 | 839300 | AT1G02340 | FBI1 | It is involved in phytochrome signaling |
| 3 | 261658_at | -1.781057411 | 5.68E-14 | 841428 | AT1G50040 | NA | Formin-like protein |
| 4 | 263480_at | -1.610126336 | 9.70E-13 | 814931 | AT2G04032 | ZIP7 | Zinc transporter 7 precursor |
| 5 | 259375_at | -4.154058844 | 4.68E-12 | 820884 | AT3G16370 | NA | GDSL-motif esterase/acyltransferase/lipase. |
| 6 | 256310_at | -2.173572939 | 9.58E-12 | 839916 | AT1G30360 | ERD4 | Early-responsive to dehydration stress protein. |
| 7 | 262980_at | -3.143308062 | 1.21E-11 | 843902 | AT1G75680 | AtGH9B7 | Glycosyl hydrolase 9B7 |
| 8 | 264978_at | -3.440808621 | 1.44E-11 | 839601 | AT1G27120 | NA | Encodes a Golgi-localized hydroxyproline-O-galactosyltransferase. |
| 9 | 256766_at | -3.279599604 | 1.47E-11 | 821788 | AT3G22231 | PCC1 | Upregulated in response to virulent and avirulent strains of *P. syringae* pv. tomato |
| 10 | 262414_at | -2.431780907 | 3.91E-11 | 841367 | AT1G49430 | LACS2 | It encodes a long chain acyl-CoA synthetase that is involved in the catalysis of Omega-hydroxy fatty acyl-CoA intermediates synthesis in the pathway of cutin synthesis |

**Supplementary Table 13. Top ten upregulated genes (ETM9C9).**

| **S.N.** | **ProbeID** | **Fold Change** | **P-value** | **EntrezID** | **TAIR Accession** | **Gene Symbol** | **Description** |
| --- | --- | --- | --- | --- | --- | --- | --- |
| 1 | 251745_at | 4.037881937 | 2.80E-14 | 824764 | AT3G55980 | ATSZF1 | Salt-inducible zinc finger 1 |
| 2 | 263403_at | 6.425970802 | 1.74E-13 | 814938 | AT2G04040 | ATDTX1 | It has been investigated as a detoxifying efflux carrier for antibiotics and other compounds derived from plants |
| 3 | 265725_at | 5.556292332 | 9.63E-13 | 817763 | AT2G32030 | NA | Acyl-CoA N-acyltransferases (NAT) superfamily protein. |
| 4 | 248686_at | 3.96512722 | 1.69E-12 | 834910 | AT5G48540 | NA | Receptor-like protein kinase-related family protein. |
| 5 | 263948_at | 7.934427724 | 3.00E-12 | 818171 | AT2G35980 | ATNHL10 | Arabidopsis non-race specific disease resistance gene (NDR1) |
| 6 | 261450_s_at | 6.053578054 | 3.39E-12 | 838707 | AT1G21110 | IGMT3 | O-methyltransferase family protein. |
| 7 | 259979_at | 4.058904574 | 5.36E-12 | 843993 | AT1G76600 | NA | Poly polymerase |
| 8 | 245082_at | 7.77620217 | 1.27E-11 | 816859 | AT2G23270 | NA | Transmembrane protein |
| 9 | 253416_at | 4.824691311 | 1.74E-11 | 829444 | AT4G33070 | AtPDC1 | Thiamine pyrophosphate dependent pyruvate decarboxylase family protein |
| 10 | 252214_at | 3.776403131 | 1.89E-11 | 824188 | AT3G50260 | ATERF#011 | Involved in defense and freezing stress responses. |

**Supplementary Table 14. Top ten upregulated genes (ETM24C24).**

| **S.N.** | **ProbeID** | **Fold Change** | **P-value** | **EntrezID** | **TAIR Accession** | **Gene Symbol** | **Description** |
| --- | --- | --- | --- | --- | --- | --- | --- |
| 1 | 245628_at | 4.847919595 | 9.50E-19 | 842120 | AT1G56650 | ATMYB75 | Interacts with JAZ proteins to regulate anthocyanin accumulation. |
| 2 | 256589_at | 4.514911338 | 8.93E-17 | 822506 | AT3G28740 | CYP81D11 | Its over-expression leads to the synthesis of volatile compounds that affect insect interactions and chemical ecology. |
| 3 | 253177_s_at | 2.780618781 | 1.99E-14 | 829667 | AT4G35150 | NA | O-methyltransferase family protein. |
| 4 | 258791_at | 4.894834971 | 6.00E-14 | 819632 | AT3G04720 | AtPR4 | It encodes a protein like the anti-fungal chitin-binding protein. Its expression is increases in response to ethylene and turnip crinkle virus infection. |
| 5 | 258957_at | 7.30051873 | 6.44E-14 | 821135 | AT3G01420 | ALPHA-DOX1 | It encodes an alpha-dioxygenase which is involved in defense against oxidative stress and cell death. It is induced in response to oxidative stress and salicylic acid |
| 6 | 245317_at | 7.58879663 | 6.98E-14 | 827238 | AT4G15610 | NA | Uncharacterized protein family. |
| 7 | 259009_at | 4.403046471 | 1.75E-13 | 820082 | AT3G09260 | BGLU23 | It is involved in the mutualistic interaction among *Arabidopsis* and *Piriformospora indica*: an endophytic fungus |
| 8 | 249154_at | 4.755306349 | 3.10E-13 | 834360 | AT5G43410 | NA | It encodes a member of ERF/AP2 TF. Its overexpression increases resistance to necrotrophic pathogen. |
| 9 | 263403_at | 6.049876876 | 5.26E-13 | 814938 | AT2G04040 | ATDTX1 | It has been investigated as a detoxifying efflux carrier for antibiotics and other compounds derived from plants |
| 10 | 256324_at | 4.050654946 | 6.45E-13 | 842994 | AT1G66760 | NA | MATE efflux family protein. |

**Supplementary Table 15. Top ten downregulated genes (ETM9C9).**

| **S.N.** | **ProbeID** | **Fold Change** | **P-value** | **EntrezID** | **TAIR Accession** | **Gene Symbol** | **Description** |
| --- | --- | --- | --- | --- | --- | --- | --- |
| 1 | 250928_at | -2.602657839 | 2.61E-09 | 831889 | AT5G03280 | ATEIN2 | Involved in ethylene signal transduction. |
| 2 | 258925_at | -2.293288092 | 1.02E-08 | 820206 | AT3G10420 | SPD1 | P-loop containing nucleoside triphosphate hydrolases superfamily protein. |
| 3 | 266078_at | -3.213130511 | 1.36E-08 | 818662 | AT2G40670 | ARR16 | Response regulator 16 |
| 4 | 252243_at | -1.107703344 | 8.63E-08 | 824174 | AT3G50120 | NA | Transmembrane protein |
| 5 | 251244_at | -1.298259801 | 2.02E-07 | 825379 | AT3G62060 | NA | Pectinacetylesterase family protein |
| 6 | 261588_at | -0.439193714 | 2.71E-07 | 839365 | AT1G01670 | NA | RING/U-box superfamily protein |
| 7 | 247746_at | -1.17303224 | 2.73E-07 | 836014 | AT5G58970 | ATUCP2 | Uncoupling protein |
| 8 | 245319_at | -1.873238379 | 3.23E-07 | 827304 | AT4G16146 | NA | cAMP-regulated phosphoprotein 19-related protein |
| 9 | 258434_at | -2.941819113 | 6.33E-07 | 820929 | AT3G16770 | ATEBP | Encodes a member of the ERF/AP2 transcription factor family (RAP2.3). |
| 10 | 253065_at | -0.72541969 | 7.05E-07 | 829930 | AT4G37740 | AtGRF2 | Growth regulating factor encoding transcription activator. |

**Supplementary Table 16. Top ten downregulated genes (ETM24C24).**

| **S.N.** | **ProbeID** | **Fold Change** | **P-value** | **EntrezID** | **TAIR Accession** | **Gene Symbol** | **Description** |
| --- | --- | --- | --- | --- | --- | --- | --- |
| 1 | 248423_at | -1.234702886 | 2.01E-28 | 835241 | AT5G51670 | NA | Hypothetical protein |
| 2 | 259417_at | -0.220667183 | 1.85E-18 | 839300 | AT1G02340 | FBI1 | Basic helix-loop-helix (bHLH) DNA-binding superfamily protein. |
| 3 | 261658_at | -1.690247549 | 1.49E-13 | 841428 | AT1G50040 | NA | Formin-like protein |
| 4 | 263480_at | -1.592859571 | 1.18E-12 | 814931 | AT2G04032 | ZIP7 | Zinc transporter 7 precursor |
| 5 | 264978_at | -3.280162808 | 3.35E-11 | 839601 | AT1G27120 | NA | Galactosyltransferase family protein |
| 6 | 250327_at | -4.40925632 | 1.02E-10 | 831078 | AT5G12050 | NA | rho GTPase-activating protein |
| 7 | 253254_at | -1.248136285 | 1.05E-10 | 829617 | AT4G34650 | SQS2 | Squalene synthase 2 |
| 8 | 247881_at | -2.81571907 | 1.07E-10 | 835877 | AT5G57700 | NA | BNR/Asp-box repeat family protein |
| 9 | 262414_at | -2.273921807 | 1.27E-10 | 841367 | AT1G49430 | LACS2 | Long-chain acyl-CoA synthetase 2 |
| 10 | 250413_at | -2.37195325 | 1.28E-10 | 830985 | AT5G11160 | APT5 | Adenine phosphoribosyltransferase 5 |

**Supplementary Table 17.** Identified up-regulated sequences in *Brassica rapa* involved in defense response against fungi using *Arabidopsis sequences* as query through BLAST analysis under contrast WTC

| **S.N.** | ***Arabidopsis thaliana***  **(TAIR Accession)** | **Length** | ***Brassica rapa***  **(PlantGDB accession)** | **Length** | **Bits Score** | **Identities** | **E-Value** |
| --- | --- | --- | --- | --- | --- | --- | --- |
| 1 | AT5G57220 | 1476 | Bra006830 | 1482 | 1674 | 87% | 0.0 |
| 2 | AT1G80840 | 909 | Bra035148 | 903 | 1026 | 87% | 0.0 |
| 3 | AT5G40990* | 1125 | - | - | - | - | - |
| 4 | AT5G46350 | 981 | Bra017561 | 966 | 933 | 84% | 0.0 |
| 5 | AT2G02930 | 639 | Bra036260 | 642 | 848 | 91% | 0.0 |
| 6 | AT2G40140 | 1794 | Bra004982 | 1671 | 931 | 84% | 0.0 |
| 7 | AT2G38470 | 1560 | Bra000064 | 1422 | 833 | 88% | 0.0 |
| 8 | AT1G19250 | 1593 | Bra031073 | 1581 | 1949 | 89% | 0.0 |
| 9 | AT2G38870 | 213 | - | - | - | - | - |
| 10 | AT5G20230 | 591 | Bra002283 | 534 | 392 | 85% | 2e-108 |
| 11 | AT2G39210 | 1806 | Bra017085 | 1767 | 1767 | 87% | 0.0 |
| 12 | AT5G64120 | 987 | Bra024269 | 987 | 1038 | 86% | 0.0 |
| 13 | AT2G37130 | 984 | Bra023099 | 993 | 1279 | 90% | 0.0 |
| 14 | AT3G52400 | 1026 | Bra012806 | 927 | 704 | 81% | 0.0 |
| 15 | AT4G38540 | 1224 | Bra033568 | 1206 | 1452 | 88% | 0.0 |
| 16 | AT5G61600 | 726 | Bra012938 | 777 | 569 | 83% | 9e-162 |
| 17 | AT2G39660 | 1188 | Bra000141 | 1182 | 1323 | 87% | 0.0 |
| 18 | AT2G30770 | 1512 | Bra022813 | 1404 | 1580 | 87% | 0.0 |
| 19 | AT3G12500 | 1008 | Bra034754 | 972 | 1037 | 87% | 0.0 |
| 20 | AT5G09440 | 837 | Bra028635 | 888 | 693 | 84% | 0.0 |
| 21 | AT1G12200 | 1398 | Bra026986 | 1395 | 1757 | 90% | 0.0 |
| 22 | AT5G47910 | 2766 | Bra037520 | 2769 | 3114 | 87% | 0.0 |
| 23 | AT4G12490 | 549 | Bra000775 | 501 | 350 | 85% | 1e-095 |
| 24 | AT4G12480 | 507 | - | - | - | - | - |
| 25 | AT3G16530 | 831 | Bra021101 | 831 | 826 | 85% | 0.0 |
| 26 | AT1G51800 | 2685 | Bra030416 | 2688 | 3306 | 89% | 0.0 |
| 27 | AT4G34460 | 1134 | Bra011536 | 1134 | 1474 | 90% | 0.0 |
| 28 | AT1G03850 | 480 | Bra015272 | 468 | 305 | 81% | 2e-082 |
| 29 | AT4G33430 | 1989 | Bra037006 | 1440 | 2013 | 92% | 0.0 |
| 30 | AT3G11340 | 1344 | Bra034848 | 3552 | 1406 | 86% | 0.0 |
| 31 | AT1G74710 | 1869 | Bra003789 | 1704 | 1914 | 88% | 0.0 |
| 32 | AT2G30020 | 1191 | Bra022772 | 1128 | 819 | 85% | 0.0 |
| 33 | AT3G01290 | 858 | Bra039130 | 861 | 1125 | 91% | 0.0 |
| 34 | AT1G02360 | 819 | - | - | - | - | - |
| 35 | AT4G01700 | 843 | Bra036316 | 840 | 1042 | 89% | 0.0 |
| 36 | AT5G39580 | 960 | Bra028436 | 957 | 952 | 85% | 0.0 |
| 37 | AT3G11820 | 1041 | Bra001422 | 1002 | 898 | 84% | 0.0 |
| 38 | AT4G12470 | 486 | - | - | - | - | - |
| 39 | AT3G49110 | 1065 | Bra029933 | 1050 | 1293 | 89% | 0.0 |
| 40 | AT3G16500 | 810 | Bra021184 | 810 | 939 | 88% | 0.0 |
| 41 | AT4G23100 | 1569 | Bra019332 | 1548 | 1930 | 89% | 0.0 |
| 42 | AT5G43580 | 300 | - | - | - | - | - |
| 43 | AT1G52400 | 1587 | Bra018969 | 1587 | 1572 | 85% | 0.0 |
| 44 | AT1G15010 | 429 | Bra016675 | 372 | 363 | 85% | 1e-099 |
| 45 | AT1G45145 | 357 | Bra014037 | 357 | 368 | 85% | 2e-101 |
| 46 | AT2G43510 | 270 | Bra004768 | 267 | 315 | 90% | 2e-085 |
| 47 | AT4G21980 | 414 | Bra019407 | 375 | 490 | 91% | 4e-138 |

***No hits found**

**Supplementary Table 18.** Identified up-regulated sequences in *Brassica rapa* involved in defense response against fungi using *Arabidopsis sequences* as query through BLAST analysis under contrast JAMC

| **S.N.** | ***Arabidopsis thaliana***  **(TAIR Accession)** | **Length** | ***Brassica rapa***  **(PlantGDB accession)** | **Length** | **Bits Score** | **Identities** | **E-Value** |
| --- | --- | --- | --- | --- | --- | --- | --- |
| 1 | AT5G57220 | 1476 | Bra006830 | 1482 | 1674 | 87% | 0.0 |
| 2 | AT1G80840 | 909 | Bra035148 | 903 | 1026 | 87% | 0.0 |
| 3 | AT4G34460 | 1134 | Bra011536 | 1134 | 1474 | 90% | 0.0 |
| 4 | AT5G40990* | 1125 | - | - | - | - | - |
| 5 | AT5G46350 | 981 | Bra017561 | 966 | 933 | 84% | 0.0 |
| 6 | AT1G03850 | 480 | Bra015272 | 468 | 305 | 81% | 2e-082 |
| 7 | AT2G02930 | 639 | Bra036260 | 642 | 848 | 91% | 0.0 |
| 8 | AT2G40140 | 1794 | Bra004982 | 1671 | 931 | 84% | 0.0 |
| 9 | AT2G38470 | 1560 | Bra000064 | 1422 | 833 | 88% | 0.0 |
| 10 | AT4G33430 | 1989 | Bra037006 | 1440 | 2013 | 92% | 0.0 |
| 11 | AT3G11340 | 1344 | Bra034848 | 3552 | 1406 | 86% | 0.0 |
| 12 | AT1G19250 | 1593 | Bra031073 | 1581 | 1949 | 89% | 0.0 |
| 13 | AT2G38870* | 213 | - | - | - | - | - |
| 14 | AT5G20230 | 591 | Bra002283 | 534 | 392 | 85% | 2e-108 |
| 15 | AT2G39210 | 1806 | Bra017085 | 1767 | 1980 | 87% | 0.0 |
| 16 | AT5G64120 | 987 | Bra024269 | 987 | 1038 | 86% | 0.0 |
| 17 | AT3G52400 | 1026 | Bra012806 | 704 | 927 | 81% | 0.0 |
| 18 | AT4G38540 | 1224 | Bra033568 | 1206 | 1452 | 88% | 0.0 |
| 19 | AT3G01290 | 858 | Bra039130 | 861 | 1125 | 91% | 0.0 |
| 20 | AT5G61600 | 726 | Bra012938 | 777 | 569 | 83% | 9e-162 |
| 21 | AT2G39660 | 1188 | Bra000141 | 1182 | 1323 | 87% | 0.0 |
| 22 | AT2G30770 | 1512 | Bra022813 | 1404 | 1580 | 87% | 0.0 |
| 23 | AT1G02360* | 819 | - | - | - | - | - |
| 24 | AT4G01700 | 843 | Bra036316 | 840 | 1042 | 89% | 0.0 |
| 25 | AT1G12200 | 1398 | Bra026986 | 1395 | 1751 | 90% | 0.0 |
| 26 | AT5G39580 | 960 | Bra028436 | 957 | 952 | 85% | 0.0 |
| 27 | AT3G11820 | 1041 | Bra001422 | 1002 | 898 | 86% | 0.0 |
| 28 | AT4G12490 | 549 | Bra000775 | 501 | 350 | 85% | 1e-095 |
| 29 | AT4G12480* | 507 | - | - | - | - | - |
| 30 | AT4G12470* | 486 | - | - | - | - | - |
| 31 | AT3G16530 | 831 | Bra021101 | 831 | 826 | 85% | 0.0 |
| 32 | AT1G51800 | 2685 | Bra030416 | 2688 | 3306 | 89% | 0.0 |
| 33 | AT4G11890 | 1065 | Bra000754 | 1077 | 846 | 81% | 0.0 |
| 34 | AT1G74710 | 1869 | Bra003789 | 1704 | 1914 | 88% | 0.0 |
| 35 | AT2G30020 | 1191 | Bra022772 | 1128 | 819 | 85% | 0.0 |
| 36 | AT3G49110 | 1065 | Bra029933 | 1050 | 1293 | 89% | 0.0 |
| 37 | AT4G23100 | 1569 | Bra019332 | 1548 | 1930 | 89% | 0.0 |
| 38 | AT1G01470* | 456 | - | - | - | - | - |
| 39 | AT5G43580* | 300 | - | - | - | - | - |
| 40 | AT2G25000 | 816 | Bra011299 | 954 | 211 | 83% | 7e-054 |
| 41 | AT2G37130 | 984 | Bra023099 | 993 | 1279 | 90% | 0.0 |
| 42 | AT2G25110 | 657 | Bra007818 | 660 | 835 | 90% | 0.0 |
| 43 | AT1G45145 | 357 | Bra014037 | 357 | 368 | 85% | 2e-101 |
| 44 | AT1G64280* | 1782 | - | - | - | - | - |
| 45 | AT5G60950* | 615 | - | - | - | - | - |
| 46 | AT3G12500 | 1008 | Bra034754 | 972 | 1037 | 87% | 0.0 |
| 47 | AT5G09440 | 837 | Bra028635 | 888 | 693 | 84% | 0.0 |
| 48 | AT5G17290 | 1014 | Bra023609 | 1026 | 1203 | 88% | 0.0 |
| 49 | AT3G56400 | 885 | Bra014692 | 858 | 673 | 83% | 0.0 |
| 50 | AT2G14560* | 624 | - | - | - | - | - |
| 51 | AT2G43510 | 270 | Bra004768 | 267 | 315 | 90% | 2e-085 |
| 52 | AT4G21980 | 414 | Bra019407 | 375 | 490 | 91% | 4e-138 |

***No hits found**

**Supplementary Table 19.** Identified up-regulated sequences in *Brassica rapa* involved in defense response against fungi using *Arabidopsis sequences* as query through BLAST analysis under contrast SAMC

| **S.N.** | ***Arabidopsis thaliana***  **(TAIR Accession)** | **Length** | ***Brassica rapa***  **(PlantGDB accession)** | **Length** | **Bits Score** | **Identities** | **E-Value** |
| --- | --- | --- | --- | --- | --- | --- | --- |
| 1 | AT5G57220 | 1476 | Bra006830 | 1482 | 1674 | 87% | 0.0 |
| 2 | AT1G80840 | 909 | Bra035148 | 903 | 1026 | 87% | 0.0 |
| 3 | AT5G40990* | 1125 | - | - | - | - | - |
| 4 | AT5G46350 | 981 | Bra017561 | 966 | 933 | 84% | 0.0 |
| 5 | AT2G02930 | 639 | Bra036260 | 642 | 848 | 91% | 0.0 |
| 6 | AT2G40140 | 1794 | Bra004982 | 1671 | 931 | 84% | 0.0 |
| 7 | AT2G38470 | 1560 | Bra000064 | 1422 | 833 | 88% | 0.0 |
| 8 | AT2G38870* | 213 | - | - | - | - | - |
| 9 | AT5G20230 | 591 | Bra002283 | 534 | 392 | 85% | 2e-108 |
| 10 | AT5G64120 | 987 | Bra024269 | 987 | 1038 | 86% | 0.0 |
| 11 | AT3G52400 | 1026 | Bra012806 | 927 | 704 | 81% | 0.0 |
| 12 | AT4G38540 | 1224 | Bra033568 | 1206 | 1452 | 88% | 0.0 |
| 13 | AT2G30020 | 1191 | Bra022772 | 1128 | 819 | 85% | 0.0 |
| 14 | AT5G61600 | 726 | Bra012938 | 777 | 569 | 83% | 9e-162 |
| 15 | AT2G39660 | 1188 | Bra000141 | 1182 | 1323 | 87% | 0.0 |
| 16 | AT2G30770 | 1512 | Bra022813 | 1404 | 1580 | 87% | 0.0 |
| 17 | AT3G12500 | 1008 | Bra034754 | 972 | 1037 | 87% | 0.0 |
| 18 | AT5G09440 | 837 | Bra028635 | 888 | 693 | 84% | 0.0 |
| 19 | AT1G12200 | 1398 | Bra026986 | 1395 | 1751 | 90% | 0.0 |
| 20 | AT5G47910 | 2766 | Bra037520 | 2769 | 3114 | 87% | 0.0 |
| 21 | AT5G39580 | 960 | Bra028436 | 957 | 952 | 85% | 0.0 |
| 22 | AT3G16530 | 831 | Bra021101 | 831 | 826 | 85% | 0.0 |
| 23 | AT3G49110 | 1065 | Bra029933 | 1050 | 1293 | 89% | 0.0 |
| 24 | AT3G16500 | 810 | Bra021184 | 810 | 939 | 88% | 0.0 |
| 25 | AT4G23100 | 1569 | Bra019332 | 1548 | 1930 | 89% | 0.0 |
| 26 | AT5G43580* | 300 | - | - | - | - | - |
| 27 | AT1G52410 | 2280 | Bra018970 | 2106 | 1184 | 79% | 0.0 |
| 28 | AT1G52400 | 1587 | Bra018969 | 1587 | 1572 | 85% | 0.0 |
| 29 | AT2G37130 | 984 | Bra023099 | 993 | 1279 | 90% | 0.0 |
| 30 | AT1G15010 | 429 | Bra016675 | 372 | 363 | 85% | 1e-099 |
| 31 | AT1G45145 | 357 | Bra014037 | 357 | 368 | 85% | 2e-101 |
| 32 | AT2G43510 | 270 | Bra004768 | 267 | 315 | 90% | 2e-085 |
| 33 | AT4G19230 | 1455 | Bra012551 | 1404 | 1786 | 90% | 0.0 |
| 34 | AT1G51800 | 2685 | Bra030416 | 2688 | 3306 | 89% | 0.0 |
| 35 | AT4G34460 | 1134 | Bra011536 | 1134 | 1474 | 90% | 0.0 |
| 36 | AT4G34410 | 807 | Bra017656 | 813 | 521 | 80% | 3e-147 |
| 37 | AT4G33430 | 1989 | Bra037006 | 1440 | 2013 | 92% | 0.0 |
| 38 | AT2G34930 | 2718 | Bra005378 | 2715 | 2516 | 84% | 0.0 |
| 39 | AT3G01290 | 858 | Bra039130 | 861 | 1125 | 91% | 0.0 |
| 40 | AT4G01700 | 843 | Bra036316 | 840 | 1042 | 89% | 0.0 |
| 41 | AT1G05800 | 1416 | Bra015454 | 1422 | 1530 | 86% | 0.0 |
| 42 | AT3G11820 | 1041 | Bra001422 | 1041 | 898 | 86% | 0.0 |
| 43 | AT4G12490* | 549 | - | - | - | - | - |
| 44 | AT4G12480* | 507 | - | - | - | - | - |
| 45 | AT4G12470 | 486 | Bra000775 | 501 | 429 | 83% | 1e-119 |

***No hits found**

**Supplementary Table 20.** Identified up-regulated sequences in *Brassica rapa* involved in defense response against fungi using *Arabidopsis sequences* as query through BLAST analysis under contrast ETMC

| **S.N.** | ***Arabidopsis thaliana***  **(TAIR Accession)** | **Length** | ***Brassica rapa***  **(PlantGDB accession)** | **Length** | **Bits Score** | **Identities** | **E-Value** |
| --- | --- | --- | --- | --- | --- | --- | --- |
| 1 | AT5G57220 | 1476 | Bra006830 | 1482 | 1674 | 87% | 0.0 |
| 2 | AT1G80840 | 909 | Bra035148 | 903 | 1026 | 87% | 0.0 |
| 3 | AT5G40990* | 1125 | - | - | - | - | - |
| 4 | AT5G46350 | 981 | Bra017561 | 966 | 933 | 84% | 0.0 |
| 5 | AT1G03850 | 480 | Bra015272 | 468 | 305 | 81% | 2e-082 |
| 6 | AT2G02930 | 639 | Bra036260 | 642 | 848 | 91% | 0.0 |
| 7 | AT2G40140 | 1794 | Bra004982 | 1671 | 931 | 84% | 0.0 |
| 8 | AT2G38470 | 1560 | Bra000064 | 1422 | 833 | 88% | 0.0 |
| 9 | AT4G33430 | 1989 | Bra037006 | 1440 | 2013 | 92% | 0.0 |
| 10 | AT1G19250 | 1593 | Bra031073 | 1581 | 1949 | 89% | 0.0 |
| 11 | AT2G38870* | 213 | - | - | - | - | - |
| 12 | AT5G20230 | 591 | Bra002283 | 534 | 392 | 85% | 2e-108 |
| 13 | AT2G39210 | 1806 | Bra017085 | 1767 | 1980 | 87% | 0.0 |
| 14 | AT5G64120 | 987 | Bra024269 | 987 | 1038 | 86% | 0.0 |
| 15 | AT3G52400 | 1026 | Bra012806 | 927 | 704 | 81% | 0.0 |
| 16 | AT4G38540 | 1224 | Bra033568 | 1206 | 1452 | 88% | 0.0 |
| 17 | AT2G30020 | 1191 | Bra022772 | 1128 | 819 | 85% | 0.0 |
| 18 | AT2G39660 | 1188 | Bra000141 | 1182 | 1323 | 87% | 0.0 |
| 19 | AT2G30770 | 1512 | Bra022813 | 1404 | 1580 | 87% | 0.0 |
| 20 | AT5G09440 | 837 | Bra028635 | 888 | 693 | 84% | 0.0 |
| 21 | AT1G02360* | 819 | - | - | - | - | - |
| 22 | AT1G12200 | 1398 | Bra026986 | 1395 | 1751 | 90% | 0.0 |
| 23 | AT5G47910 | 2766 | Bra037520 | 2769 | 3114 | 87% | 0.0 |
| 24 | AT5G39580 | 960 | Bra028436 | 957 | 952 | 85% | 0.0 |
| 25 | AT4G12490* | 549 | - | - | - | - | - |
| 26 | AT2G43510 | 270 | Bra004768 | 267 | 315 | 90% | 2e-085 |
| 27 | AT3G16530 | 831 | Bra021101 | 831 | 826 | 85% | 0.0 |
| 28 | AT3G49110 | 1065 | Bra029933 | 1050 | 1293 | 89% | 0.0 |
| 29 | AT3G16500 | 810 | Bra021184 | 810 | 939 | 88% | 0.0 |
| 30 | AT4G23100 | 1569 | Bra019332 | 1548 | 1930 | 89% | 0.0 |
| 31 | AT5G43580* | 300 | - | - | - | - | - |
| 32 | AT2G25000 | 816 | Bra011299 | 954 | 211 | 83% | 7e-054 |
| 33 | AT1G52410 | 2280 | Bra018970 | 2106 | 1184 | 79% | 0.0 |
| 34 | AT1G52400 | 1587 | Bra018969 | 1587 | 1572 | 85% | 0.0 |
| 35 | AT2G37130 | 984 | Bra023099 | 993 | 1279 | 90% | 0.0 |
| 36 | AT1G45145 | 357 | Bra014037 | 357 | 368 | 85% | 2e-101 |
| 37 | AT5G60950* | 615 | - | - | - | - | - |
| 38 | AT3G12500 | 1008 | Bra034754 | 972 | 1037 | 87% | 0.0 |
| 39 | AT4G19230 | 1455 | Bra012551 | 1404 | 1786 | 90% | 0.0 |
| 40 | AT1G51800 | 2685 | Bra030416 | 2688 | 3306 | 89% | 0.0 |
| 41 | AT4G34460 | 1134 | Bra011536 | 1134 | 1474 | 90% | 0.0 |
| 42 | AT3G11340 | 1344 | Bra034848 | 3552 | 1406 | 86% | 0.0 |
| 43 | AT1G74710 | 1869 | Bra003789 | 1704 | 1914 | 88% | 0.0 |
| 44 | AT3G01290 | 858 | Bra039130 | 861 | 1125 | 91% | 0.0 |
| 45 | AT5G61600 | 726 | Bra012938 | 777 | 569 | 83% | 9e-162 |
| 46 | AT4G01700 | 843 | Bra036316 | 840 | 1042 | 89% | 0.0 |
| 47 | AT3G11820 | 1041 | Bra001422 | 1002 | 898 | 86% | 0.0 |
| 48 | AT4G12480* | 507 | - | - | - | - | - |
| 49 | AT4G12470 | 486 | Bra000775 | 501 | 429 | 83% | 1e-119 |

***No hits found**
